# Cannabinoid Profiling of Hemp Seed Oil by Liquid Chromatography Coupled to High-Resolution Mass Spectrometry

**DOI:** 10.3389/fpls.2019.00120

**Published:** 2019-02-13

**Authors:** Cinzia Citti, Pasquale Linciano, Sara Panseri, Francesca Vezzalini, Flavio Forni, Maria Angela Vandelli, Giuseppe Cannazza

**Affiliations:** ^1^Department of Life Sciences, University of Modena and Reggio Emilia, Modena, Italy; ^2^CNR NANOTEC, Institute of Nanotechnology, Lecce, Italy; ^3^Department of Health, Animal Science and Food Safety, University of Milan, Milan, Italy

**Keywords:** hemp seed oil, hemp, high-resolution mass spectrometry, cannabinoids, cannabinoids mass spectra

## Abstract

Hemp seed oil is well known for its nutraceutical, cosmetic and pharmaceutical properties due to a perfectly balanced content of omega 3 and omega 6 polyunsaturated fatty acids. Its importance for human health is reflected by the success on the market of organic goods in recent years. However, it is of utmost importance to consider that its healthy properties are strictly related to its chemical composition, which varies depending not only on the manufacturing method, but also on the hemp variety employed. In the present work, we analyzed the chemical profile of ten commercially available organic hemp seed oils. Their cannabinoid profile was evaluated by a liquid chromatography method coupled to high-resolution mass spectrometry. Besides tetrahydrocannabinol and cannabidiol, other 30 cannabinoids were identified for the first time in hemp seed oil. The results obtained were processed according to an untargeted metabolomics approach. The multivariate statistical analysis showed highly significant differences in the chemical composition and, in particular, in the cannabinoid content of the hemp oils under investigation.

## Introduction

*Cannabis sativa* L. is one of the most widespread cultivations in the world, well known for its characteristic to produce a class of terpenophenolic compounds named phytocannabinoids (Elsohly and Slade, 2005). According to the most recent cannabinoid inventory, at least 120 phytocannabinoids have been identified to date (Hanuš et al., 2016). They can be divided into 11 subclasses depending on their chemical structure: cannabigerol (CBG-type), (–)-Δ^9^-tetrahydrocannabinol (Δ^9^-THC-type), cannabidiol (CBD-type), cannabichromene (CBC-type), cannabinol (CBN-type), (–)-Δ^8^-tetrahydrocannabinol (Δ^8^-THC-type), cannabicyclol (CBL-type), cannabinodiol (CBND-type), cannabielsoin (CBE-type), cannabitriol (CBT-type) and miscellaneous type (Elsohly and Slade, 2005). For long time neutral phytocannabinoids have been considered as the actual products of cannabis inflorescence (Hanuš et al., 2016). Actually, the fresh plant produces the acidic form of phytocannabinoids, thus it is now accepted that the neutral forms derive from the non-enzymatic decarboxylation of their acidic counterpart. It is necessary to underline that many phytocannabinoids that have been isolated so far are artifacts generated by non-enzymatic reactions occurring either in the plant or during the analytical processes for their identification (Hanuš et al., 2016).

The two main phytocannabinoids produced by cannabis are CBD and THC. Whilst the latter is an intoxicating substance, the former is completely void of the “high” effects of its isomer THC (Mechoulam et al., 2002). On the other hand, CBD has proved to have several pharmacological properties, thus ranking among the most studied phytocannabinoids for its possible therapeutic use in a number of pathologies (Pisanti et al., 2017). Depending on the variety of cannabis plant, it can produce predominantly either THC or CBD. It has been suggested to distinguish cannabis between drug-type (marijuana) and fiber-type (hemp), the former being high in THC and the latter high in CBD. This classification is based on the intoxicating effect of THC (Small, 2015). However, considering the recent use of CBD as a drug, it should be more appropriate to distinguish cannabis between THC-type and CBD-type. Furthermore, breeders have recently selected a number of cannabis varieties, popularly called “industrial hemp,” that predominantly produce CBG (de Meijer and Hammond, 2005). Therefore, a CBG-type should be added to the list. All these phytocannabinoids are produced in the glandular trichomes, which contains a resin oil mainly made of phytocannabinoids and terpenes (Small, 2015). Such glandular bodies are present essentially on the female flowering and fruiting tops of cannabis plant and their highest concentration is measured on the bracts, the two small leaves surrounding the seed (Small, 2015).

Hemp seed oil is becoming popular in Italy as well as in other countries due to the healthy properties associated to the perfectly balanced fatty acid composition that meet the FAO/WHO recommendations (Food and Agriculture Organization [FAO]/World Health Organization [WHO], 2008). While being void of cannabinoids in the inside, seeds can be contaminated on the outer surface by the sticky resin oil secreted by the numerous glandular trichomes present on the bracts (Ross et al., 2000). As a result, the surface of the seed will be “dirty” with all the cannabinoids present in the resin oil of that specific cannabis variety. As the seeds are employed mainly for oil production, if they are cleaned properly prior to the extraction of hemp seed oil, the latter will contain only traces of cannabinoids. Conversely, it has been recently suggested that some commercial hemp seed oils can carry a total THC concentration above 10 ppm and total CBD over 1000 ppm (Citti et al., 2018c). Therefore, cannabis variety and the seed cleaning procedures affect, respectively the qualitative and quantitative profile of all cannabinoids eventually present in the hemp seed oil. In this view, it is reasonable to hypothesize that other cannabinoids might be present in the hemp seed oil. Since each cannabinoid is responsible for a specific pharmacological activity (Izzo et al., 2009), it is of utmost importance to define the cannabinoid profile of any commercially available hemp seed oil. For instance, if the oil were produced from CBG-type cannabis, we would expect to find a predominant concentration of CBG, thus the oil should have specific nutraceutical properties exerted by this cannabinoid. Finola and Futura, CBD-rich hemp varieties, are listed in the European cannabis varieties for industrial purposes and are indicated as the varieties of choice for hemp oil production due to the discrete amount of seeds produced (Galasso et al., 2016).

A number of works in the literature report the determination of THC and CBD concentration in hemp seed oil (Bosy and Cole, 2000; Leizer et al., 2000; Lachenmeier et al., 2004), but, to the best of our knowledge, there is no study regarding the evaluation of the comprehensive cannabinoid profile in this cannabis product.

Our research group, and more recently other groups (Berman et al., 2018; Calvi et al., 2018), has developed liquid chromatography methods coupled to high-resolution mass spectrometry detection (HPLC-HRMS) for the identification of the different cannabinoids in cannabis medicinal extracts based on both exact mass and match of the fragmentation pattern (MS^2^) of pure analytical standards of the known cannabinoids. Exploiting HRMS technique, it is possible to define the comprehensive cannabinoid profile in commercial hemp seed oils in order to address their different nutraceutical properties to a specific cannabinoid. The present work is indeed focused on the identification and semi-quantification of the main and best-known cannabinoids in commercially available hemp seed oils, CBD and THC, along with other “minor” cannabinoids, which contribute to the final beneficial effects. A multivariate statistical analysis (MSA) was also carried out to highlight the significant differences among the commercial hemp seed oils.

## Materials and Methods

### Chemicals and Reagents

All solvents (acetonitrile, water, 2-propanol, formic acid) were LC-MS grade and purchased from Carlo Erba (Milan, Italy). Certified analytical standards of CBGA, THCA, CBDA, CBDV, Δ^9^-THC, Δ^8^-THC, CBD, Δ^9^-THC-*d_3_*, CBD-*d_3_*, CBG, CBC and CBN were purchased from Cerilliant (Sigma-Aldrich, Round Rock, Texas). Organic hemp seed oils were bought from the Italian market and numbered from Oil_1 to Oil_10.

### Preparation of Standard Solutions and Hemp Seed Oil Samples

Stock solutions of CBDV, CBDA, CBGA, CBG, CBD, CBN, Δ^9^-THC, Δ^8^-THC, CBC and THCA (1000 μg/mL) in methanol were diluted in blank matrix to the final concentration of 10 μg/mL. An aliquot of 100 μL of each sample was diluted with 890 μL of blank matrix and 10 μL of IS (Δ^9^-THC-*d_3_* and CBD-*d_3_*, 200 μg/mL) to the final concentration of 1 μg/mL for CBDV, CBDA, CBGA, CBG, CBD, CBN, Δ^9^-THC, Δ^8^-THC, CBC and THCA and 2 μg/mL for IS.

For the semi-quantification of the identified cannabinoids, the stock solution of the analytical standards mixture was diluted with blank matrix to the final concentrations of 0.01, 0.05, 0.10, 0.25, 0.50, 0.75, and 1.00 μg/mL.

Blank matrix was obtained as described in our previous work (Citti et al., 2018c). Briefly, 22 g of hemp seeds (cleared of bracts) were washed with ethyl alcohol 96% (3 × 100 mL) in order to remove cannabinoids. Subsequently, the seeds were cold squeezed to obtain 4 mL of hemp seed oil where the level of cannabinoids was below the limit of detection. The final blank matrix (20 mL) was obtained by diluting the oil with 16 mL of 2-propanol.

Authentic samples were obtained by diluting 100 μL of hemp seed oil with 395 μL of 2-propanol and 5 μL of IS working solution.

Quality control samples (QCs) were prepared to assess the reliability of the statistical model by mixing a 10 μL aliquot from each oil sample. QCs were analyzed in triplicate at the beginning of the batch and every 10 runs.

### UHPLC-HRMS/MS Analyses

LC analyses were performed on an Ultimate 3000 UHPLC ultrahigh performance liquid chromatograph (Thermo Fisher Scientific, San Jose, CA, United States), consisting of a vacuum degasser, a quaternary pump, a thermostated autosampler and a thermostated column compartment. The sampler temperature was set at 15°C and the column compartment temperature at 25°C. A Poroshell 120 EC-C18 column (3.0 × 100 mm, 2.7 μm, Agilent, Milan, Italy) was used to separate the compounds of interest with a mobile phase composed of 0.1% formic acid in both (A) water and (B) acetonitrile. The gradient elution was set as follows: 0.0–45.0 min linear gradient from 5 to 95% B; 45.1–55.0 min 95% B; 55.1–60.0 min back to 5% B and equilibration of the column for 5 min. The total run time was 65 min. The flow rate was set at 0.3 mL/min. The sample injection volume was 5 μL.

The UHPLC system is interfaced to a Q-Exactive Plus mass spectrometer (Thermo Fisher Scientific, San Jose, CA, United States) equipped with a heated electrospray ionization (HESI) source. The optimized parameters were as follows: capillary temperature, 320°C; vaporizer temperature, 280°C; electrospray voltage, 4.2 kV (positive mode) and 3.8 kV (negative mode); sheath gas, 55 arbitrary units; auxiliary gas, 30 arbitrary units; S lens RF level, 45. Analyses were carried out using Xcalibur 3.0 software (Thermo Fisher Scientific, San Jose, CA, United States). The exact masses of the compounds were calculated using Qual Browser in Xcalibur 3.0 software. All Q-Exactive parameters (RP, AGC and IT) were optimized by direct infusion of cannabinoid analytical standards (10 μg/L) with a flow rate of 0.1 mL/min in order to improve sensitivity and selectivity. The analyses were acquired in FS-dd-MS^2^ (full scan data-dependent acquisition) in positive and negative mode separately at a resolving power of 70,000 FWHM at *m/z* 200. The scan range was set at *m/z* 250–400 improving the sensitivity of detection; the automatic gain control (AGC) was set at 3e6, with an injection time of 100 ms. The isolation window of the quadrupole that filters the precursor ions was set at *m/z* 2. Fragmentation of precursors was optimized at four values of normalized collision energy (NCE) (20, 30, 40, and 50 eV) by injecting working mix standard solution at a concentration of 10 μg/L. Detection was based on calculated [M+H]^+^ and [M–H]^-^ molecular ions with an accuracy of 2 ppm, retention time and fragments match (*m/z* and intensity).

### Data Processing and Multivariate Statistical Analysis

Raw LC-HRMS/MS data were processed using XCMS Online platform (Gowda et al., 2014). In particular, the platform applies peak detection, retention time correction, profile alignment, and isotope annotation. The raw files were organized in datasets and processed as a multi-group type experiment. The parameters were set as follows: centWave for feature detection (Δ*m/z* = 5 ppm, minimum and maximum peak width 5 and 40 s, respectively); obiwarp settings for retention time correction (profStep = 1); parameters for chromatogram alignment, including mzwid = 0.025, minfrac = 0.5, and bw = 5. The relative quantification of the identified compounds was based on the corresponding peak areas. Metabolite identification was based on accurate mass (within 2 ppm) and/or MS^2^ data match against MS^2^ spectra of compounds available on mzCloud database (HighChem LLC, Slovakia). The results output was exported and processed with MetaboAnalyst 3.0 for MSA (Xia and Wishart, 2016). Principal component analysis (PCA) was obtained after data normalization by a specified feature (CBD-*d_3_*) and autoscaling. Partial Least Square Discriminant Analysis (PLS-DA) was performed to maximize the groups difference. One-way ANOVA test was performed setting the adjusted *p*-value cut-off at 0.01 and using the Tukey’s honest Significant Difference *post hoc* test. A heatmap was built according to Euclidean distance and Ward clustering algorithm on normalized and auto-scaled data.

## Results

### LC-HRMS Analysis and Mass Fragmentation Characterization

**FIGURE 1:**
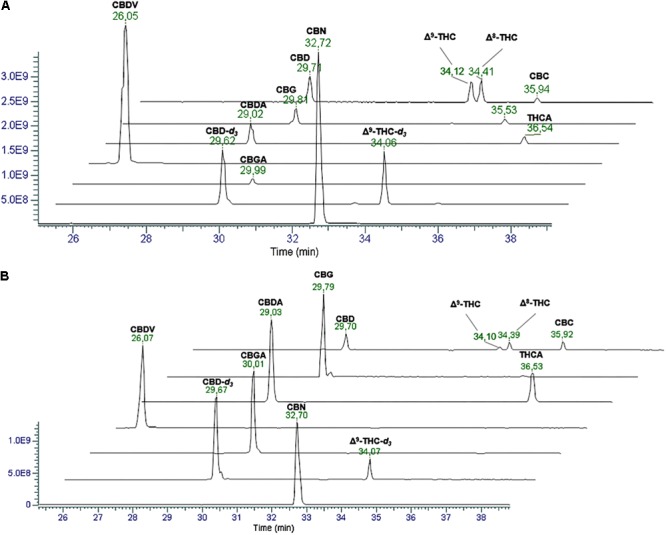
Extracted Ion Chromatograms (EICs) in positive **(A)** and negative **(B)** ionization mode of a mix solution of cannabinoid standards (1 μg/mL). From the top: CBD, Δ^9^-THC and Δ^8^-THC ([M+H]^+^ 315.2319, [M–H]^-^ 313.2173), CBG ([M+H]^+^ 317.2475, [M–H]^-^ 315.2330), CBDA and THCA ([M+H]^+^ 359.2217, [M–H]^-^ 357.2071), CBDV ([M+H]^+^ 287.2006, [M–H]^-^ 285.1860), CBGA ([M+H]^+^ 361.2373, [M–H]^-^ 359.2228), internal standards (IS) (2 μg/mL) CBD-*d_3_* and THC-*d_3_* ([M+H]^+^ 318.2517, [M–H]^-^ 313.2361), and CBN ([M+H]^+^ 311.2006, [M–H]^-^ 309.1860).

The first goal of the present work was to develop a chromatographic method able to separate the different cannabinoids. In particular, since most of them are isomers and show similar fragmentation spectra, their identification is possible only according to their retention time. A chromatographic method for the chemical profiling of cannabis oil medicinal extracts has been previously developed by our group (Citti et al., 2018a). This method has been adapted to the purpose of the present work and proved to be suitable for the separation of cannabinoids in hemp seed oil. The separation of the compounds of interest was carried out on a core-shell stationary phase in reverse phase mode, which showed good performances in terms of retention of the analytes, peak shape and resolution power (Citti et al., 2016a,b, 2018a,b,c,d). A gradient elution was used starting from low percentages of the organic modifier (5% acetonitrile) to 95% in 45 min. This allowed for an optimal separation of cannabinoids from minute 18.0 of the chromatographic run. Figure 1 reports the extracted ion chromatograms (EIC) in positive (A) and negative (B) mode of a cannabinoid standard mixture at 1 μg/mL used to assess the reliability of the chromatographic method. The separation between CBDA and CBGA, CBD and CBG does not represent an issue when working with MS detection since there is a 2.0156 amu difference between the two cannabinoids. Conversely, the separation between Δ^9^-THC and Δ^8^-THC, which present the same molecular ion and identical fragmentation at low NCE (20), could be quite tricky. However, in this case, we were able to obtain a baseline resolution using the abovementioned chromatographic conditions.

Since very few works in the literature describe the fragmentation mechanism of the most common cannabinoids using an electrospray ionization source in both positive and negative mode, the first part of the work regarded the elucidation of the fragmentation patterns of the precursor ions [M+H]^+^ and [M–H]^-^ of the cannabinoid standards (CBDA, CBGA, THCA, CBDV, CBD, CBG, CBN, Δ^9^-THC, Δ^8^-THC and CBC). In order to propose a reliable fragmentation mechanism, we exploited the mass spectra of the cannabinoid deuterated standards.

#### Cannabidiol-Type

In the LC-MS chromatogram, CBD elutes after its acidic precursor CBDA due to its higher lipophilicity. On the other end, shorter alkyl chain homologs, like CBDV, elute before CBDA and CBD due to lower lipophilicity.

**FIGURE 2:**
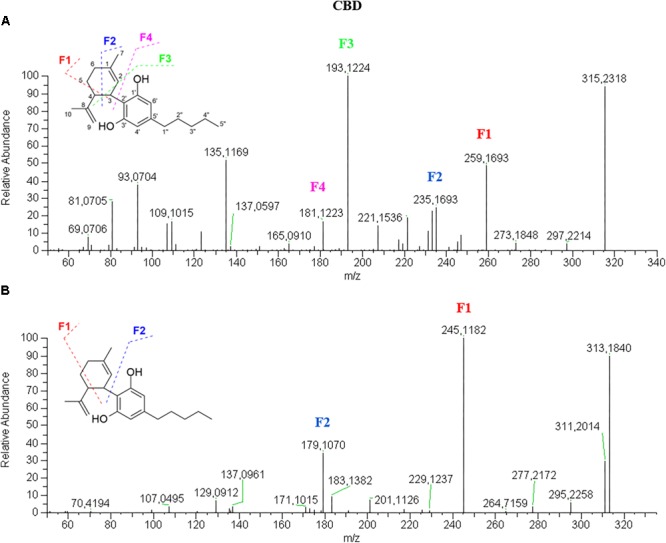
HRMS fragmentation spectrum of cannabidiol (CBD) in positive **(A)** and negative **(B)** ionization mode.

In positive mode, as shown in Figure 2A, CBD [M+H]^+^ molecular ion 315.2318 (90% relative abundance) presents a fragment-rich spectrum, the most relevant of which are: 259.1693 (50%) deriving from the loss of four carbon units from the terpene moiety; 235.1693 (30%) corresponding to the breakage of the terpene with only four carbon units of this moiety left; 193.1224, which is the base peak (100%), corresponding to olivetol with the carbon unit attached to C2 of the benzene ring; and 181.1223 (20%) corresponding to the resorcinol moiety (olivetol in this specific case). Furthermore, a fragment with *m/z* 135.1169, which is constant in most cannabinoid fragmentations in positive mode, corresponds to the terpene moiety. It might be easy to misinterpret the fragmentation mechanism as a neutral loss of 56 that generates the fragment 259 can be also obtained by breaking the side alkyl chain at the 1”–2” bond. However, this breakage is more difficult to occur than that on the terpene moiety. Moreover, the fragmentation spectrum of CBD-*d_3_* shows the presence of the three deuterium atoms in the fragments 262.1892, 238.1890, 210.1562, 196.1420 and 184.1420. This suggests that all the fragments are originated from the bond breakage on the terpene moiety since the deuterium atoms are on C5′′ of the alkyl chain. The presence of the fragment 135 in the CBD-*d_3_* spectrum confirmed the proposed mechanism. In negative mode (Figure 2B), CBD molecular ion [M–H]^-^ 313.2172 (90%) generates a limited number of fragments, the most abundant of which are 245.1545 (100%), originated from the retro Diels-Alder and 179.1068 (40%) corresponding to the olivetol moiety. This fragmentation mechanism was confirmed by the MS/MS spectrum of CBD-*d_3_* in negative mode (Supplementary Figure S1).The acidic precursor CBDA (Supplementary Figure S2) shows a main fragment with *m/z* 341.2110 (100%) in positive mode obtained from the loss of H_2_O (–18). The [M+H]^+^ molecular ion 359.2213 is barely visible. The other relevant fragments are 261.1485 (10%) and 219.1015 (10%), which are obtained from the breakage of the terpene moiety at C1–C6 bond and from the terpene loss (with only C3 left), respectively. In negative mode, CBDA molecular ion [M–H]^-^ 357.2072 (100%) generates two fragments with *m/z* 339.1965 (70%) and with *m/z* 313.2173 consequent to the loss of a molecule of water and CO_2_, respectively, producing the CBD molecule (30%). Besides the fragments 245.1545 (20%) and 179.1068 (25%), also present in the CBD spectrum, a retro Diels-Alder reaction occurs on the molecule after the loss of water generating the fragment 271.1341 (10%).Fragmentation spectra of CBDV (Supplementary Figure S6) in both positive and negative ionization mode are consistent with its pentyl homolog CBD with a 28 amu difference (corresponding to a (–CH_2_)_2_). Likewise, the intensity of all fragments in the CBDV spectrum is identical to that of the fragments in the CBD spectrum.

#### Tetrahydrocannabinol-Type

Δ^9^- and Δ^8^-THC elute after CBD and CBN due to the loss of a free hydroxyl group and the formation of the dihydropyran ring, which confers higher lipophilicity. The chromatographic conditions employed allows an optimal separation of the two isomers, which is important when the MS spectrum does not help with the identification. Basically, no difference can be highlighted between Δ^9^-THC and Δ^8^-THC in either positive or negative ionization mode at NCE of 20 (Supplementary Figure S11). However, the literature reports that the two molecules can be distinguished in negative mode at NCE above 40 by the intensity of the product ion 191.1070 with respect to the precursor ion 313.2172 (Berman et al., 2018).

**FIGURE 3:**
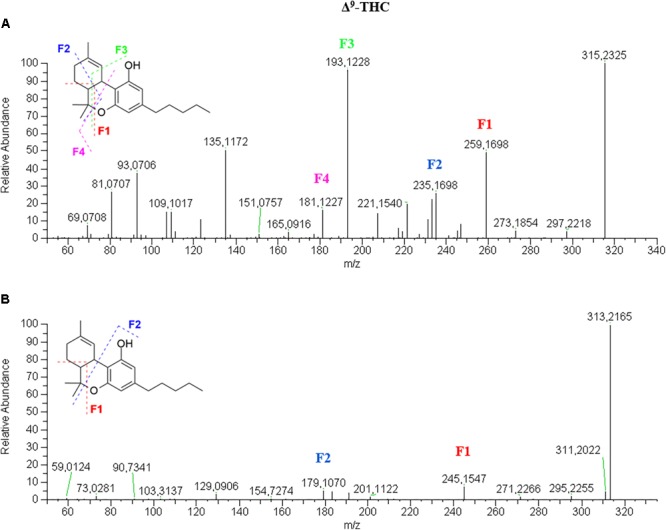
HRMS fragmentation spectrum of Δ^9^-tetrahydrocannabinol (Δ^9^-THC or THC) in positive **(A)** and negative **(B)** ionization mode.

Δ^9^-THC spectrum in positive mode (Figure 3A) is very similar to that of CBD. In this case, only the retention time can be indicative of the identity of the molecule. On the other hand, the fragmentation pattern in negative mode (Figure 3B) shows a great difference in terms of number of fragments. THC appears less fragmented than CBD as the fragments 245.1544 and 179.1068 show intensities below 10% and the molecular ion [M–H]^-^ 313.2172 is the base peak. The fragmentation mechanism was elucidated by the analysis of Δ^9^-THC-*d_3_* spectra (Supplementary Figure S12).

The same consideration could be made for the acidic precursor THCA (Supplementary Figure S13), which shows a fragmentation spectrum in positive mode similar to that of CBDA to the point that they could be easily mistaken. Conversely, the fragmentation of THCA in negative mode shows only a major peak at *m/z* 313.2173 (45%) corresponding to the loss of CO_2_ to generate the “neutral” derivative THC. The loss of water leads to a very small fragment 339.1962 (5%), which is probably more unstable that the corresponding species obtained with CBDA. The dihydropyran ring probably confers different chemical properties and reactivity to the whole molecule. Moreover, the acidic species elutes after the neutral counterpart, opposite to the case of CBDA/CBD.

#### Cannabinol-Type

CBN elutes after CBD because of the additional pyran ring, which confers higher lipophilicity, but before THC due to the presence of aromaticity responsible for a higher polarity compared to the simple cyclohexane.

**FIGURE 4:**
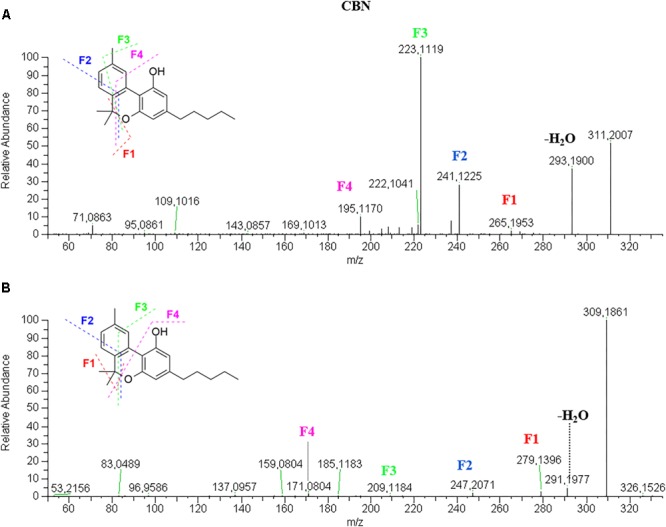
HRMS fragmentation spectrum of cannabinol (CBN) in positive **(A)** and negative **(B)** ionization mode.

In positive mode (Figure 4A), CBN molecular ion [M+H]^+^ 311.2006 (64%) shows a product ion at 293.1895 (40%) given by the loss of water, another one at 241.1220 (30%) due to the benzopyran ring opening, the base peak at 223.1115, which keeps three carbon atoms of the ring, and the fragment 195.1167 (15%) corresponding to the resorcinol moiety and one carbon atom. In negative mode (Figure 4B), CBN fragmentation spectrum is very simple with only very low-intensity product ions and the molecular ion [M–H]^-^ 309.1860, which is also the base peak. It originates the fragment 279.1388 given by the pyran ring opening and loss of the two methyl groups, the fragments 247.2071 and 209.1184 due to the progressive breakage of the benzopyran ring, and the fragment 171.0806 due to the breakage of the benzene ring of the olivetol moiety. Such fragmentation does not occur in other cannabinoids most likely because the C–C bond between two benzene rings is stronger and more difficult to break than the C–C bond between a benzene ring and a terpene moiety.

#### Cannabigerol-Type

CBG elutes very close to CBD, as well as CBGA elutes immediately after CBDA. This could be explained by the slightly higher lipophilicity of the open isoprenoid chain compared to the closed limonene moiety.

**FIGURE 5:**
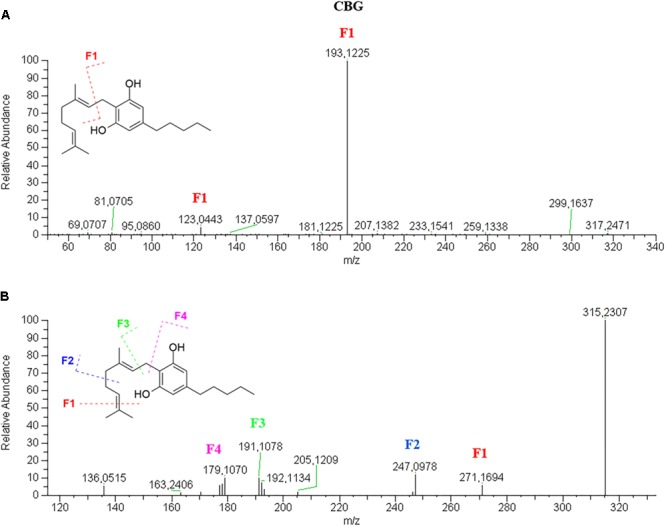
HRMS fragmentation spectrum of cannabigerol (CBG) in positive **(A)** and negative **(B)** ionization mode.

CBG has a very simple fragmentation spectrum in both positive and negative mode. The molecular ion [M+H]^+^ 317.2469 is barely visible and readily breaks to give the only product ion and base peak 193.1225, corresponding to the olivetol moiety with the ortho-methyl group (Figure 5A). The molecular ion [M–H]^-^ 315.2394, which is also the base peak, is so stable that the fragments 271.1694, 247.0978, 191.1070 and 179.1068, have very low abundance (Figure 5B). These product ions derive from the progressive loss of carbon units of the isoprenoid moiety.

The [M+H]^+^ molecular ion 361.2373 of the acidic counterpart CBGA (Supplementary Figure S20) is not stable and readily loses a molecule of water to give the ion 343.2279 (75%), which is then broken at C1–C2 of the isoprenoid moiety to give the fragment 219.1023 (100%). The [M–H]^-^ molecular ion 359.2230 (45%) generates only two main fragments, 341.2122 (100%) and 315.2329 (35%), as a result of the loss of water and CO_2_, respectively. The other fragments have very low abundance: 297.2223 (<5%) derives from the additional loss of water and 191.1069 (<5%) is in common with the neutral derivative CBG.

#### Cannabichromene-Type

CBC elutes after THC due to a ring opening and the presence of an additional long alkyl chain on the pyran ring. Its retention time is slightly lower than that of THCA.

**FIGURE 6:**
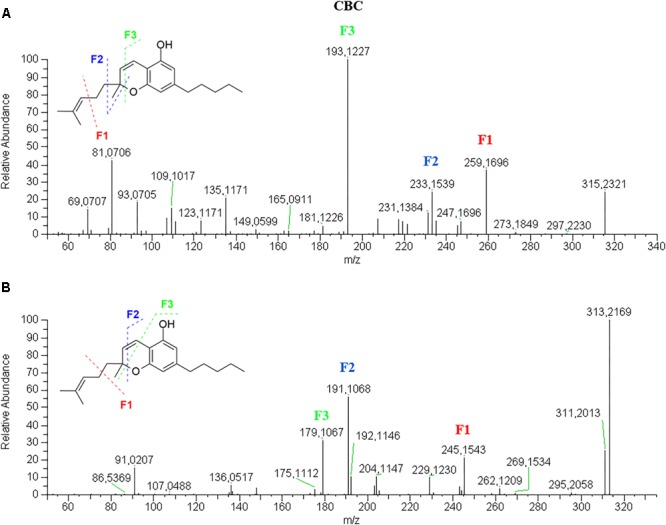
HRMS fragmentation spectrum of cannabichromene (CBC) in positive **(A)** and negative **(B)** ionization mode.

CBC has a fragmentation pattern in positive mode very similar to THC so that they are quite undistinguishable (Figure 6A). In negative mode (Figure 6B), it is possible to discriminate CBC from THC by the ionic abundance of the fragments. Like THC, the molecular ion [M–H]^-^ 313.2171 is the base peak, but unlike THC it generates a higher product ion 245.1544 (25%) deriving from the loss of one isoprene unit. The other two product ions, 191.1068 (55%) and 179.1068 (35%), are higher in CBG than THC, where they are below 10%.

### Identification of Cannabinoids in Hemp Seed Oil

Hemp seed oil is an invaluable source of nutrients and other compounds with undeniable nutraceutical properties, spanning polyunsaturated fatty acids, polyphenols, tocopherols, proteins, carbohydrates, lignanamides and cannabinoids, which contribute to the overall health benefits of this functional food (Giorgi et al., 2013; Crescente et al., 2018). While most of these classes of compounds have been thoroughly characterized, the attention on the cannabinoid class has been focused only on the major and best known of them like CBD, THC and CBN. One of our recent work extended the study to the quantification of CBG and CBDV, with particular attention to the acidic form of CBD and THC, CBDA and THCA, which are the predominant species found in cold-pressed hemp seed oil (Citti et al., 2018c). However, a comprehensive cannabinoid profile has never been defined.

**FIGURE 7:**
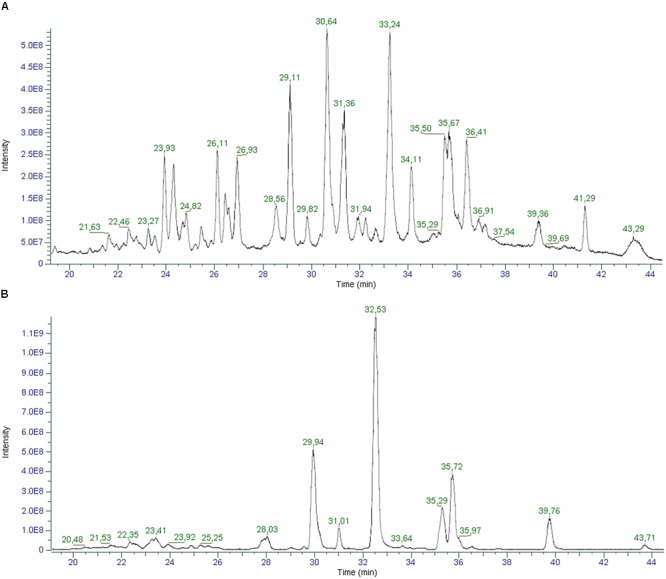
Total ion Chromatograms (TICs) of a hemp seed oil sample (oil_1) in positive **(A)** and negative **(B)** ionization mode.

In light of the new pharmacological properties ascribed to other cannabinoids different from the two main ones, THC and CBD, it is crucial to evaluate their presence in the most consumed cannabis derived food product, hemp seed oil (Hanuš et al., 2016). To this aim, we employed the cutting-edge technology for liquid chromatography and high-resolution mass spectrometry, which ensures a superior level of mass accuracy and allowed for the identification of a greater number of compounds compared to other techniques (Citti et al., 2018b). Figure 7 shows an example of the total ion chromatograms of a hemp seed oil sample obtained in positive (A) and negative (B) ionization mode.

In the present work, we report the identification of 32 cannabinoids in 10 commercial hemp seed oils obtained by organic farming. Of these, 9 cannabinoids were identified with level 1 annotation, using the corresponding analytical standards, and 23 were putatively identified with level 2 annotation, according to exact mass and mass fragmentation match with standards found in the database mzCloud and/or reported in the literature (Salek et al., 2013). It is noteworthy that for the first time a number of cannabinoids, which to the best of our knowledge have never been reported, have been identified in hemp seed oil.

A list of cannabinoids was prepared according to recently published works (Hanuš et al., 2016; Berman et al., 2018). The LC-HRMS chromatograms were screened in order to find the corresponding [M+H]^+^ and [M–H]^-^ molecular ions. A recent work by Berman et al. (2018) reports the mass fragmentation spectra in negative mode of a series of cannabinoids detected in extracts of the aerial part of cannabis plant. This helped in the selection of 15 cannabinoids which showed a perfect match of the fragmentation spectrum in negative ionization mode (cannabitriolic acid (CBTA), cannabitriol (CBT), CBGA-C_4_, CBDA-C_1_, CBDVA, CBDA-C_4_, cannabidiolic acid monomethyl ether (CBDMA), cannabielsoinic acid (CBEA), cannabinolic acid (CBNA), THCA-C_1_, tetrahydrocannabidivarin (THCV), tetrahydrocannabidivarinic acid (THCVA), THCA-C_4_, cannabichromevarin (CBCV), cannabichromevarinic acid (CBCVA)). Except for CBTA, CBGA-C_4_ and CBEA, the corresponding fragmentation spectrum in positive ionization mode has been extracted for each cannabinoid. Moreover, four other cannabinoids were added to the spectral mass library. Cannabiripsol (CBR) was identified according to its similarity with CBT as they differ only for the presence of a double bond on the latter. 6,7-Epoxy-CBG and its acidic precursor 6,7-epoxy-CBGA share the same fragmentation pattern as all CBG-type cannabinoids. Cannabicitran (CBCT) was identified based on the mass fragmentation match in mzCloud. CBD-C_1_, CBD-C_4_ THC-C_4_ and CBCT were identified according to the fragmentation spectrum obtained in positive mode as no fragmentation was observed in negative mode. All the identified cannabinoids with the corresponding chemical formula, retention time and molecular ions [M+H]^+^ and [M–H]^-^ are listed in Table 1.

**Table 1:** Cannabinoids identified in commercial hemp seed oil.

Class	Cannabinoid	R*_T_* (min)	Formula	[M+H]^+^	[M–H]^-^
Cannabiripsol (CBR)	CBR	19.27	C_21_H_32_O_4_	349.2373	347.2228
Cannabitriol (CBT)	CBTA	19.41	C_22_H_28_O_6_	391.2115	389.1970
	CBT	21.91	C_21_H_28_O_4_	347.2217	345.2071
Cannabigerol (CBG)	6,7-Epoxy-CBGA	21.25	C_22_H_32_O_5_	377.2323	375.2177
	6,7-Epoxy-CBG	24.41	C_21_H_32_O_3_	333.2424	331.2279
	CBGA-C_4_	28.10	C_21_H_30_O_4_	347.2217	345.2071
	CBGA	29.60	C_22_H_32_O_4_	361.2373	359.2228
	CBG	29.77	C_21_H_32_O_2_	317.2475	315.2330
Cannabidiol (CBD)	CBDA-C_1_	22.88	C_18_H_22_O_4_	303.1591	301.1445
	CBDVA	25.44	C_20_H_26_O_4_	331.1904	329.1758
	CBD-C_1_	25.75	C_17_H_22_O_2_	259.1693	257.1547
	CBDV	26.17	C_19_H_26_O_2_	287.2006	285.1860
	CBDA-C_4_	26.99	C_21_H_28_O_4_	345.2060	343.1915
	CBD-C_4_	27.99	C_20_H_28_O_2_	301.2162	299.2017
	CBDA	28.56	C_22_H_30_O_4_	359.2217	357.2071
	CBD	29.81	C_21_H_30_O_2_	315.2319	313.2173
	CBDMA	33.76	C_23_H_32_O_4_	373.2373	371.2228
Cannabielsoin (CBE)	CBEA	29.27	C_23_H_32_O_4_	375.2166	373.2020
Cannabinol (CBN)	CBN	32.65	C_21_H_26_O_2_	311.2006	309.1860
	CBNA	33.92	C_22_H_26_O_4_	355.1904	353.1758
Tetrahydrocannabinol (THC)	THCA-C_1_	28.12	C_18_H_22_O_4_	303.1591	301.1445
	THCV	29.92	C_19_H_26_O_2_	287.2006	285.1860
	THCVA	31.38	C_20_H_26_O_4_	331.1904	329.1758
	THC-C_4_	32.05	C_20_H_28_O_2_	301.1803	299.2017
	THCA-C_4_	33.46	C_21_H_32_O_4_	345.2060	343.1915
	THC	34.09	C_21_H_30_O_2_	315.2319	313.2173
	THCA	35.50	C_22_H_30_O_4_	359.2217	357.2071
Cannabichromene (CBC)	CBCV	31.27	C_19_H_26_O_2_	287.2006	285.1860
	CBCVA	32.58	C_20_H_26_O_4_	331.1904	329.1758
	CBC	35.19	C_21_H_30_O_2_	315.2319	313.2173
	CBCA	36.41	C_22_H_30_O_4_	359.2217	357.2071
Cannabicitran (CBCT)	CBCT	33.15	C_21_H_30_O_2_	315.2319	313.2173

*For each cannabinoid, the class, retention time (min), chemical formula and precursor ions ([M+H]^+^ and [M–H]^-^) are indicated.*

Δ^8^-THC was not detected in any of the hemp seed oil samples. Although it derives from acid- or oxidatively promoted shift of the endocyclic double bond of Δ^9^-THC and is presented as more thermodynamically stable than its precursor (Hanuš et al., 2016), the chemical environment of hemp seed oil might not be favorable for this isomerization.

Mass fragmentation spectra in positive and negative mode are reported in the Supplementary Material and are available for other researchers with similar instrumental equipment who need a possible comparison for the identification of unknown cannabinoids. A plausible fragmentation mechanism in both polarities is also proposed (Supplementary Material).

Lastly, a semi-quantification was carried out in order to provide approximate concentrations of the identified cannabinoids, since absolute quantification is applicable only to level 1 cannabinoids, for which authentic standards are available. Absolute quantification of cannabinoids from level 2 to 4^1^ is not viable without appropriate analytical ploys. Hence, the concentrations of level 1 cannabinoids (CBDA, THCA, CBGA, CBD, Δ^9^-THC, CBC, CBDV, CBN and CBG) were calculated by external calibration of authentic standards analyzed in the same LC-MS conditions. The linear equations for these cannabinoids are reported in the Supplementary Material. For level 2 cannabinoids, for which analytical standards were not available, we employed the calibration curve of the cannabinoid standard with the closest structural similarity. For those acid cannabinoids with no structural similarity, the calibration curve was set as the average ion response obtained for the same concentration for all the available acid cannabinoid standards. The same was applied to level 2 neutral cannabinoids, though leaving CBDV and CBN out as they displayed completely different ion responses most likely due to shorter alkyl chain and additional aromatization, respectively. The results of the semi-quantification are reported in Table 2.

**Table 2:** Semi-quantification of the identified cannabinoids.

Class	Cannabinoid	Oil 1	Oil 2	Oil 3	Oil 4	Oil 5	Oil 6	Oil 7	Oil 8	Oil 9	Oil 10
CBG	CBGA	0.04	0.04	0.03	0.05	0.03	0.08	0.07	0.02	0.16	0.05
	CBG	0.04	0.02	0.02	0.02	0.03	0.04	0.04	0.02	0.02	0.03
	CBGA-C_4_^1^	0.04	0.07	0.00	0.00	0.00	0.00	0.00	0.01	0.00	0.00
	6,7-Epoxy-CBGA^1^	0.00	0.11	0.06	0.00	0.00	0.00	0.00	0.02	0.00	0.02
	6,7-Epoxy-CBG^2^	0.01	0.03	0.03	0.02	0.01	0.01	0.01	0.02	0.02	0.01
CBD	CBDA	0.62	7.75	7.68	1.19	0.81	0.93	1.04	5.29	1.37	5.76
	CBD	0.08	1.08	1.53	0.24	0.12	0.11	0.14	1.01	0.26	1.37
	CBDA-C_4_^3^	0.08	0.07	0.08	0.00	0.00	0.00	0.00	0.03	0.00	0.06
	CBD-C_4_^4^	0.05	0.04	0.01	0.77	0.22	0.82	0.81	0.02	0.85	0.03
	CBDVA^3^	0.06	0.16	0.13	0.13	0.00	0.11	0.11	0.08	0.25	0.09
	CBDV	0.25	0.25	0.29	0.50	0.08	0.27	0.26	0.19	0.71	0.27
	CBDA-C_1_^3^	0.00	0.19	0.23	0.00	0.00	0.00	0.00	0.07	0.00	0.09
	CBD-C_1_^4^	0.02	0.02	0.01	0.04	0.01	0.02	0.03	0.01	0.07	0.01
	CBDMA^3^	0.07	0.00	0.00	0.21	0.07	0.19	0.22	0.00	0.31	0.00
THC	THCA	0.64	0.30	0.43	2.84	0.69	1.41	1.00	0.50	0.36	0.49
	THC	0.11	0.02	0.04	0.16	0.07	0.11	0.12	0.02	0.27	0.03
	THCA-C_4_^5^	0.00	0.00	0.00	0.02	0.00	0.00	0.03	0.00	0.23	0.00
	THC-C_4_^6^	0.06	0.00	0.00	0.04	0.00	0.00	0.14	0.19	0.37	0.01
	THCVA^5^	0.62	0.00	0.00	0.89	0.67	1.16	1.12	0.1	1.85	0.06
	THCV^6^	0.38	0.01	0.00	0.58	0.25	0.49	0.51	0.00	0.98	0.02
	THCA-C_1_^5^	0.05	0.00	0.00	0.12	0.09	0.18	0.18	0.00	0.41	0.00
CBC	CBCA^7^	0.02	0.07	0.04	0.03	0.01	0.01	0.03	0.04	0.07	0.05
	CBC	0.60	1.18	1.60	1.03	0.29	0.47	0.53	0.96	1.68	1.41
	CBCVA^7^	0.00	0.00	0.00	0.12	0.02	0.10	0.01	0.00	0.12	0.00
	CBCV^8^	0.01	0.00	0.00	0.14	0.00	0.05	0.05	0.00	0.21	0.05
CBN	CBNA^7^	0.07	0.01	0.00	0.12	0.03	0.09	0.10	0.02	0.26	0.03
	CBN	0.17	0.05	0.54	0.26	0.07	0.10	0.11	0.05	0.61	0.08
CBE	CBEA^7^	0.02	0.02	0.10	0.08	0.03	0.04	0.07	0.03	0.00	0.06
CBT	CBTA^7^	0.00	0.00	0.00	0.18	0.10	0.14	0.05	0.00	0.06	0.00
	CBT^9^	0.00	0.00	0.04	0.16	0.03	0.13	0.02	0.02	0.01	0.04
CBR	CBR^9^	0.01	0.18	0.00	0.01	0.06	0.07	0.14	0.05	0.00	0.00
CBCT	CBCT^9^	0.00	0.12	0.13	0.00	0.00	0.00	0.00	0.09	0.01	0.10

*Values are expressed in microgram per milliliter as mean of three analyses. ^*1*^For the semi-quantification of these cannabinoids, the calibration curve of CBGA was employed. ^*2*^The calibration curve employed is that of CBG. ^*3*^The calibration curve employed is that of CBDA. ^*4*^The calibration curve employed is that of CBD. ^*5*^The calibration curve employed is that of THCA. ^*6*^The calibration curve employed is that of THC. ^*7*^The calibration curve employed is obtained by the average ion response for the same concentration for all standard acid cannabinoids available (CBGA, CBDA, THCA). ^*8*^The calibration curve employed is that of CBC. ^*9*^The calibration curve employed is obtained by the average ion response for the same concentration for all standard neutral pentyl cannabinoids available (CBD, Δ^*9*^-THC, CBC, CBG).*

### Untargeted Metabolomics for Cannabinoid Profile in Hemp Seed Oil

**FIGURE 8:**
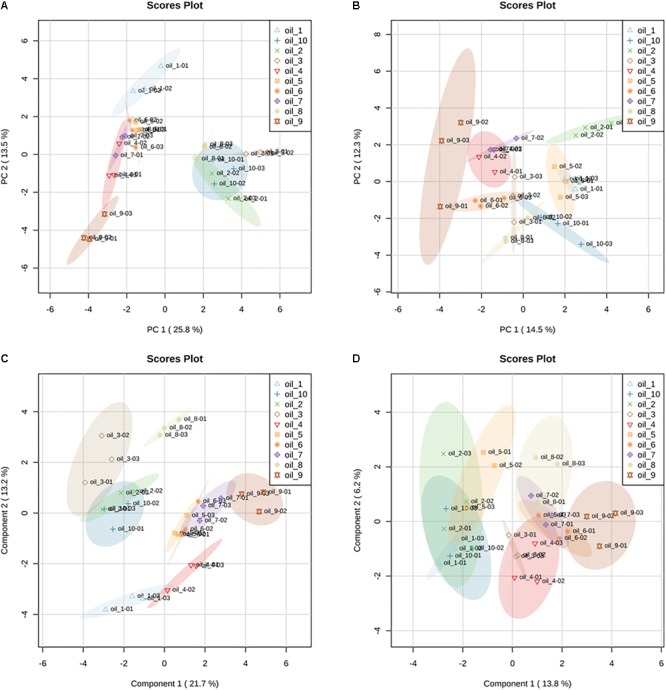
Principal Component Analysis (PCA) in positive **(A)** and negative **(B)** ionization mode of LC-HRMS data of hemp seed oils. Samples are named as “oil_number” (e.g., oil_1); the colored ellipsoids represent the 95% confidence region. Partial Least Squares Discriminant Analysis (PLS-DA) in positive **(C)** and negative **(D)** ionization mode of the LC-HRMS data of hemp seed oils. PLS-DA is performed by rotating the PCA components in order to obtain the maximum separation among the groups. Validation parameters: *R*^*2*^ = 0.915; *Q*^*2*^ = 0.755.

**FIGURE 9:**
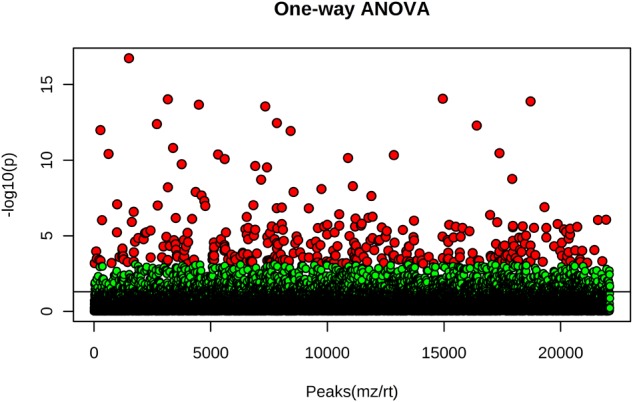
One-way ANOVA test of the ten hemp seed oil samples. Red points indicate statistically significant features, green points indicate features that do not contribute to the statistical difference (adjusted *p*-value cut-off: 0.01, *post hoc* test: Tukey’s Honest Significant Difference test).

The ten hemp seed oil samples analyzed by LC-HRMS in FS-dd-MS^2^ were processed by XCMS Online platform according to an untargeted metabolomics approach. Untargeted metabolomics was performed in order to highlight possible differences in the chemical profile among the ten samples. The results output was then processed with MetaboAnalyst 3.0, which provided the MSA. In particular, the PCA in both positive and negative mode (Figure 8A,B, respectively) showed a defined cluster organization of the different groups, which results sharpened in the Partial Least Square Discriminant Analysis (PLS-DA) (Figure 8C,D). Such separation suggests that the chemical composition of the different hemp seed oils is different. In order to address the differences, we used the PCA loadings list provided by MetaboAnalyst that indicates which variables have the largest effect on each component. Loadings close to –1 and 1 (anyway far from 0), were chosen as those that strongly influenced the clusters separation. By analyzing the spectral data, it was possible to identify several compounds, such as glucosides (sucrose, isohamnentin, *p*-coumaric acid hexoside), flavonoids (*N*-caffeoyltyramine, *N*-coumaroyltyramine, *N*-feruloyltyramine isomer 1 and 2, kampferol, cannflavin B), acids (linolenic acid, oleic acid, α-linolenic acid) and cannabinoids. Figure 9 shows all the significant features (in red) responsible for PCA clustering.

**FIGURE 10:**
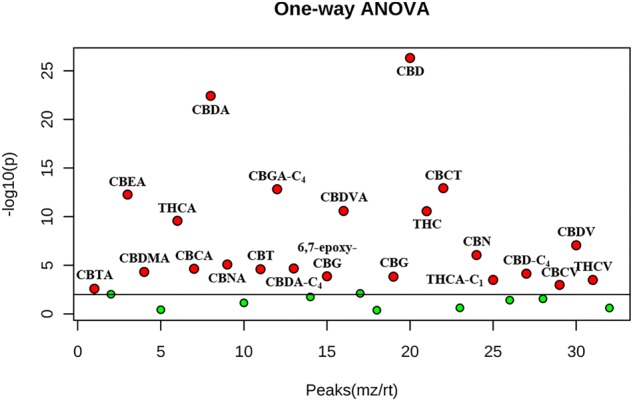
One-way ANOVA test of the ten hemp seed oil samples limited to the selected cannabinoids. Red points indicate statistically significant features, green points indicate features that do not contribute to the statistical difference (adjusted *p*-value cut-off: 0.01, *post hoc* test: Tukey’s Honest Significant Difference test).

**FIGURE 11:**
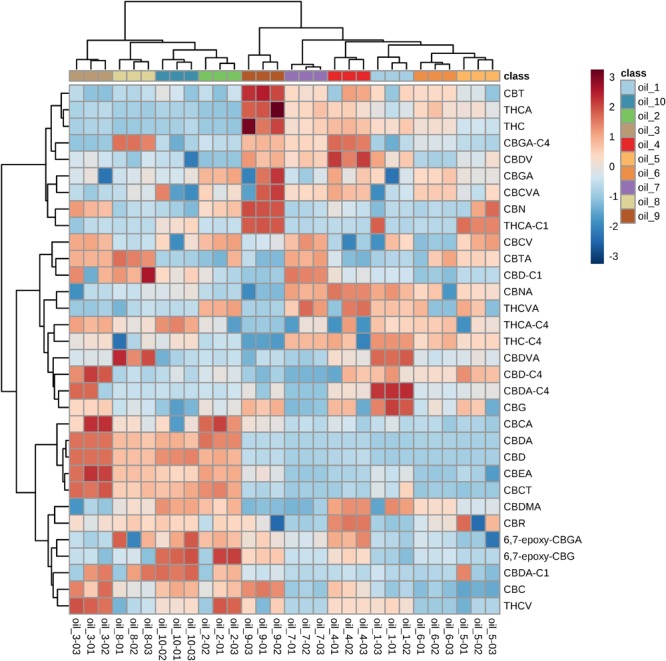
Heatmap built with the identified cannabinoids. Color-coding consists of shades of red and blue, where higher intensity of red stands for very high concentration and higher intensity of blue stands for very low concentration. The samples are shown in colors at the top of the heatmap, while cannabinoids are reported on each row.

We focused the attention on the cannabinoid group selecting those previously identified by HRMS. With one-way ANOVA test we were able to select only the statistically significant features among all the identified cannabinoids that contribute to determine the group distribution. Figure 10 displays in red the significant features and in green those that determine no difference among the ten groups. Specifically, 22 cannabinoids out of 32, CBD, CBDA, CBGA-C_4_, CBEA, CBCT, CBDVA, THC, THCA, CBDV, CBN, CBMA, CBCA, CBDA-C4, CBTA, CBNA, CBT, 6,7-epoxy-CBG, CBG, THCA-C_1_, CBD-C_4_, CBCV and THCV, ranked as statistically significant, thus contributing to the clustering of the oils along with other abovementioned important compounds. A direct picture of the distribution of significant cannabinoids over the ten samples is given in Figure 11, which represents a heatmap of the selected data.

## Discussion

Hemp seed oil has been an inestimable source of nutrients for thousands of years (Callaway, 2004). Nowadays, despite the scientific evidence that claims beneficial biological properties for this cannabis derived food product, people are still skeptical about its nutritional and therapeutic value, generally due to the potential risk ascribed to intoxicating cannabinoids (Crescente et al., 2018). However, taking into account that there are strict laws on THC levels in cannabis derived products, it is of great importance to shed lights on the beneficial effects deriving from the contribution of other cannabinoids. Indeed, it is now a common belief that either THC or CBD alone are less effective than a combination of cannabinoids or of cannabinoids and other compounds in producing the final biological activity of hemp seed oil and other cannabis derived products (Crescente et al., 2018).

For the first time several cannabinoids have been detected in hemp seed oil, most of which resulted relevant in determining a statistical difference in the chemical composition. Although CBDA and CBD rank first in determining the largest effect on the chemical differences among the ten oils due to their higher abundance, 20 other “minor” cannabinoids are also responsible for the chemical differentiation.

This adds a new question mark on the extreme variability in the chemical composition of hemp seed oil mostly deriving from the hemp variety, which is unavoidably translated to the pharmacological versatility of this product. In this context, it is important to underline that very little is known about the pharmacological activities of many cannabinoids, including cannabielsoin (CBE), CBD, THC and CBG derivatives, or CBD, THC and CBG homologs with different length of the side alkyl chain.

In fact, whilst many works report the anti-inflammatory, anti-oxidant, anti-epileptic properties of CBD (Costa et al., 2007; Pisanti et al., 2017), the anticonvulsant properties of CBN (Karler et al., 1973), the anti-inflammatory and anticancer activity of CBG (Deiana, 2017), the antibacterial properties of CBC (Turner and Elsohly, 1981), very little is known about the acidic species of cannabinoids except for CBDA, which has proved to have anticancer (Takeda et al., 2012, 2017) and antiemetic properties (Bolognini et al., 2013).

In this view, it is extremely important to bear in mind the big difference between the acidic and neutral form of a cannabinoid. For example, while THC is known for its psychotropic activity, the very few studies available in the literature suggest that THCA is void of such effects given its presumed inability to pass the blood-brain barrier (Jung et al., 2009; Guillermo, 2016), but it has shown some anti-proliferative/pro-apoptotic activity (Ligresti et al., 2006). Several studies have explored the conversion kinetics of THCA into THC, indicating that heat is required for this reaction to occur and that uncomplete conversion is unavoidably obtained at temperatures below 160°C (Perrotin-Brunel et al., 2011; Wang et al., 2016). Therefore, if hemp seed oil is consumed without heating, the levels of THC will remain low and its acidic form will be taken.

Although cannabinoids represent a small percentage among all hemp seed oil components (proteins, carbohydrates, fatty acids, etc.), the results obtained by MSA suggest they actively contribute to the chemical variability of the final product. Taking into account that each cannabinoid is responsible for a specific biological activity, it is reasonable to hypothesize that they participate to the overall effect generated by hemp seed oil consumption.

Although a semi-quantification should be regarded with different levels of confidence given the lack of analytical standards for most of the known cannabinoids, it still represents a useful tool for determining which cannabinoid is more likely to produce a biological effect. Nonetheless, the results of the semi-quantification indicated that all cannabinoids levels were below 5 ppm, considered the THC limit recommended by the German legislation, which is the most restrictive. Such low concentrations could have relevant nutraceutical effects, but it is difficult to determine the actual pharmacological evidence given the limited scientific studies regarding the minimum effective dose of cannabinoids. Apart from THC, there are no guidelines concerning the maximum daily dose of the known cannabinoids that can be consumed by a single person.

Moreover, previous works have reported that even consuming low-THC hemp seed oil, bioaccumulation and subsequent metabolite excretion may result in positive cannabinoid test in urines (Callaway et al., 1997; Lehmann et al., 1997; Struempler et al., 1997; Bosy and Cole, 2000). This consideration is applicable to all “classical” and “minor,” intoxicating and non-intoxicating cannabinoids, including those with unknown biological activity.

This scenario is further complicated since all cannabinoids generally interact with each other and/or with other non-cannabinoid compounds determining an unpredictable final effect (Morales et al., 2017; Turner et al., 2017). Hence, the relative proportions between cannabinoids are also important for the final resulting effect. At this regard, our results clearly indicate extreme variability in the cannabinoid composition between all samples. It is then expected that this variability is translated into a completely variable nutraceutical profile.

For this reason, even though it is not possible to explain the extreme pharmacological versatility arisen from the combination of all cannabinoids, the analysis and identification of as many of them as possible in each hemp seed oil sample is crucial for exploiting the full potential for human life and well-being of this unique food product.

## Ethics Statement

This study was carried out according to the authorization released to GC by Ministry of Health (SP/056, protocol number) for the supply and detention of analytical standards of narcotic drugs and/or psychotropic substances for scientific purposes.

## Author Contributions

CC and GC collaborated to the conception and design of the study, performed the statistical analysis, and coordinated the whole work. PL contributed to the experimental part and drafted the manuscript. FF and MV contributed to the experimental design and manuscript draft. SP and FV drafted the manuscript. All authors contributed to manuscript revision, read and approved the submitted version.

## Conflict of Interest Statement

The authors declare that the research was conducted in the absence of any commercial or financial relationships that could be construed as a potential conflict of interest.

## Footnotes

1 As indicated by Salek et al. (2013), compounds identified with level 1 of confidence are those whose identity is confirmed by comparing at least two chemical properties of authentic standards with the experimental data; compounds reported with level 2 of confidence are those putatively annotated; level 3 of confidence refers to putatively characterized classes of compounds; level 4 of confidence includes all unknown compounds.
