# Supplementary material for: Cannabinoid Profiling of Hemp Seed Oil by Liquid Chromatography Coupled to High-Resolution Mass Spectrometry
Source: Front Plant Sci. 2019 Feb 13;10:120. doi: 10.3389/fpls.2019.00120 (PMC6381057; doi:10.3389/fpls.2019.00120)
Supplement: Supplementary file 1 [file Data_Sheet_1.docx]

Supplementary Material

Cannabinoid profiling of hemp seed oil by liquid chromatography coupled to high-resolution mass spectrometry

Cinzia Citti^1,2*^, Pasquale Linciano^1^, Sara Panseri^3^, Francesca Vezzalini^1^, Flavio Forni^1^, Maria Angela Vandelli^1^, Giuseppe Cannazza^1,2*^

^1^Department of Life Sciences, University of Modena and Reggio Emilia, Modena, Italy

^2^CNR NANOTEC, Institute of Nanotechnology, Lecce, Italy

^3^Department of Health, Animal Science and Food Safety, University of Milan, Milan, Italy

*** Correspondence:**Cinzia Citti

cinzia.citti@unimore.it

Giuseppe Cannazza
giuseppe.cannazza@unimore.it

## HRMS spectra


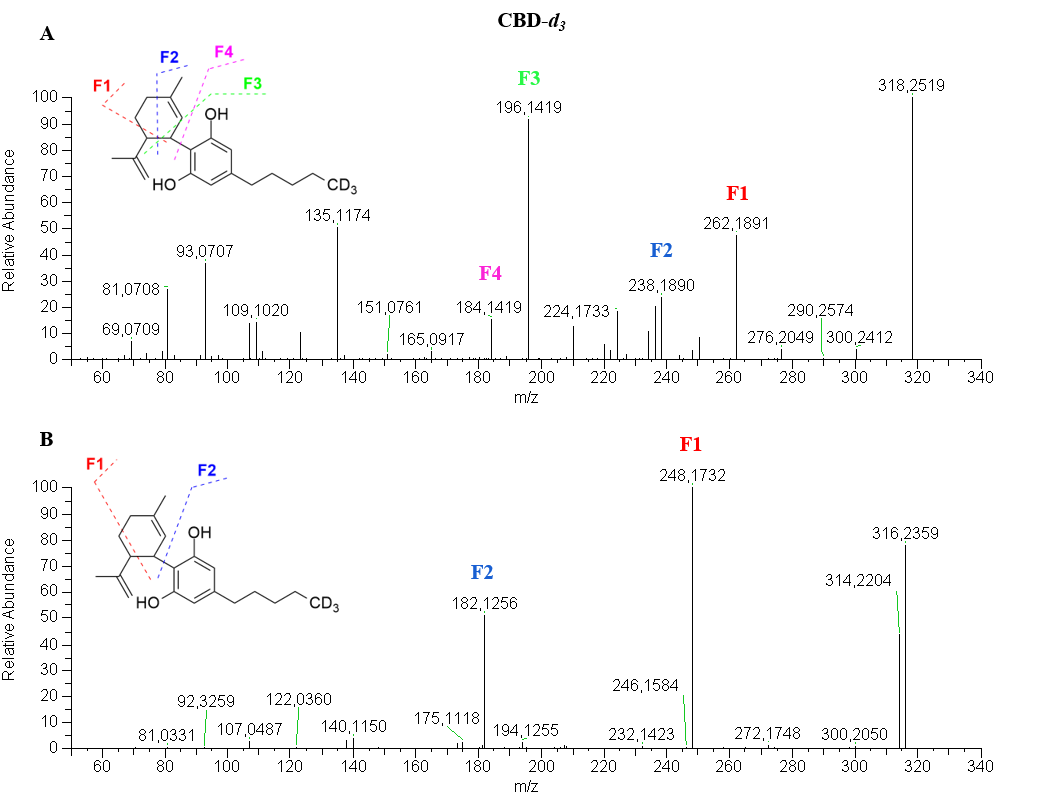


Figure S 1. HRMS fragmentation spectrum of cannabidiol-*d_3_* (CBD-*d_3_*) in positive (A) and negative (B) ionization mode.


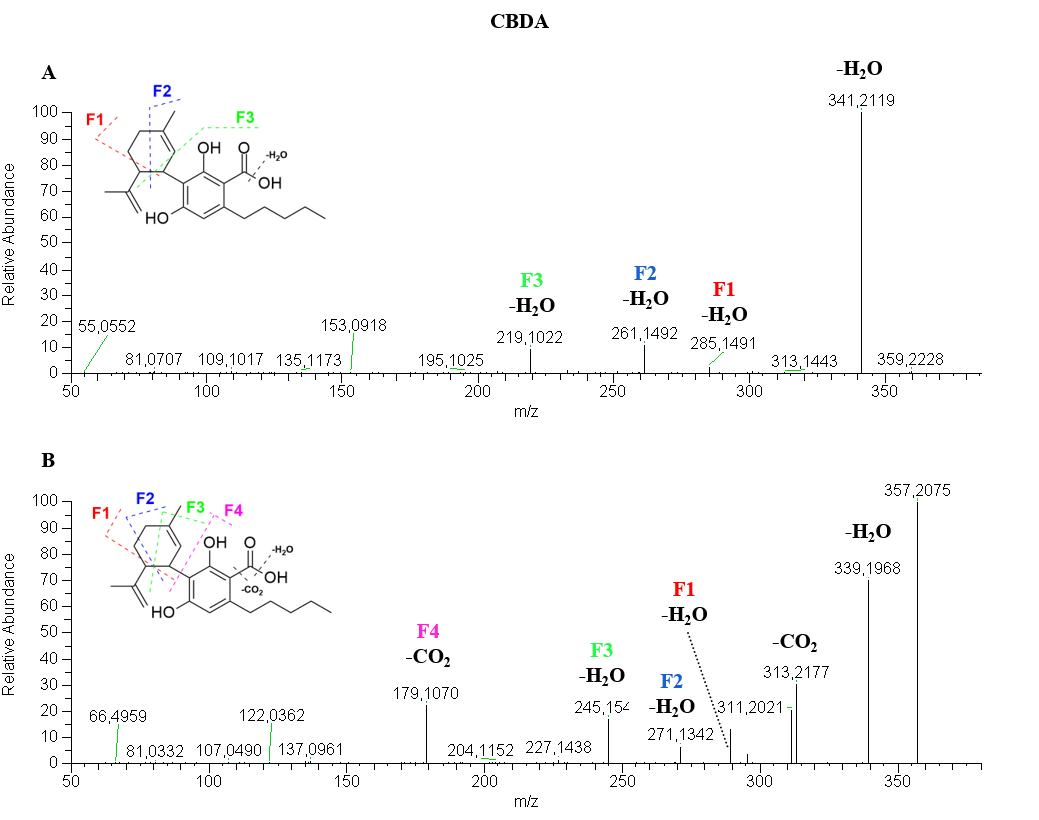


Figure S 2. HRMS fragmentation spectrum of cannabidiolic acid (CBDA) in positive (A) and negative (B) ionization mode.


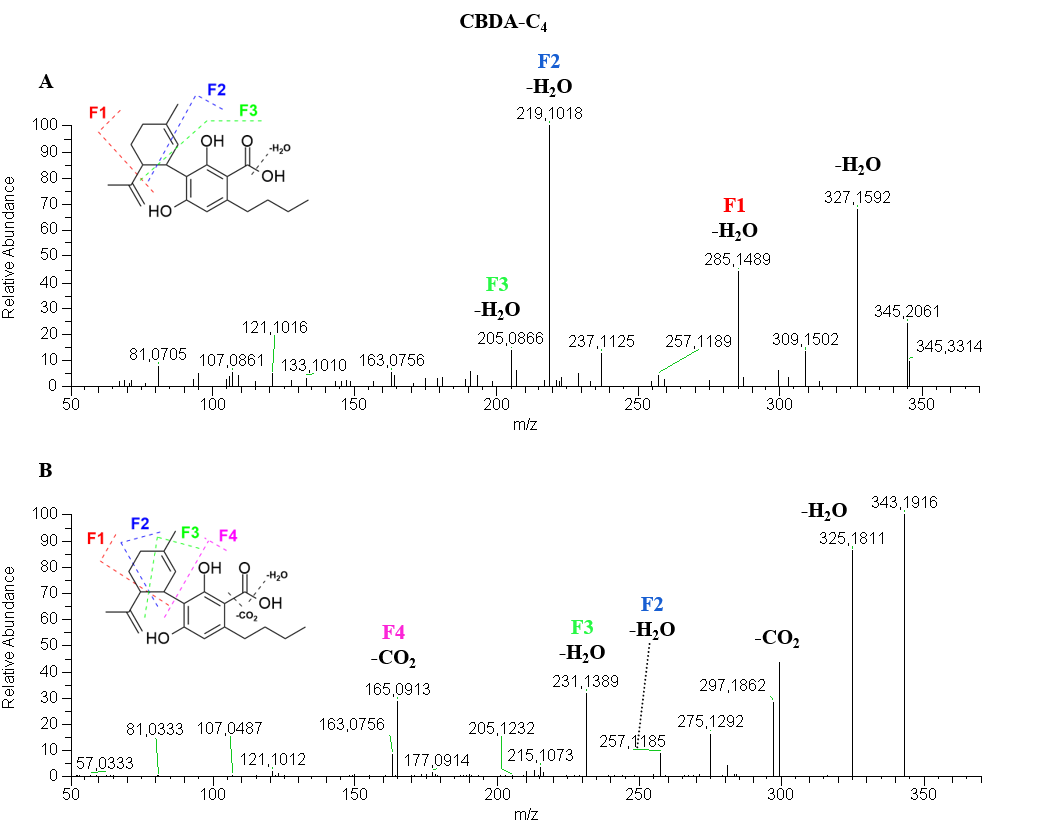


Figure S 3. HRMS fragmentation spectrum of cannabidiolic acid-C_4_ (CBDA-C_4_) in positive (A) and negative (B) ionization mode.


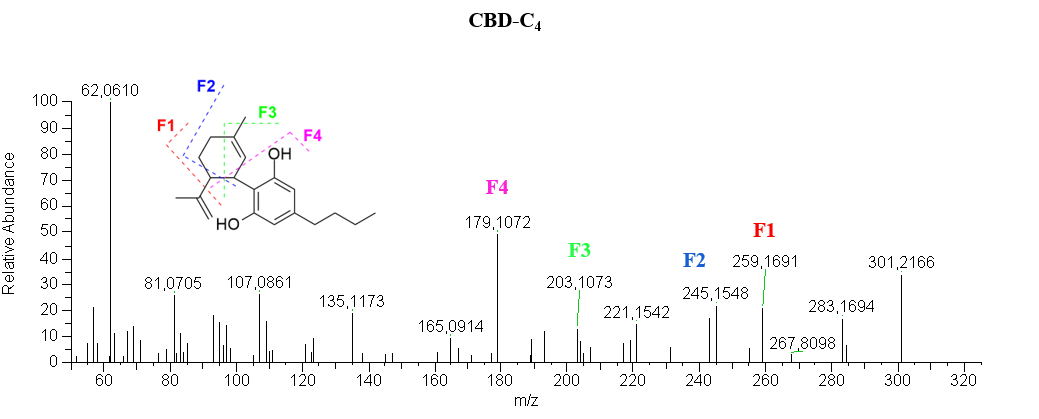


Figure S 4. HRMS fragmentation spectrum of cannabidiol-C_4_ (CBD-C_4_) in positive ionization mode.


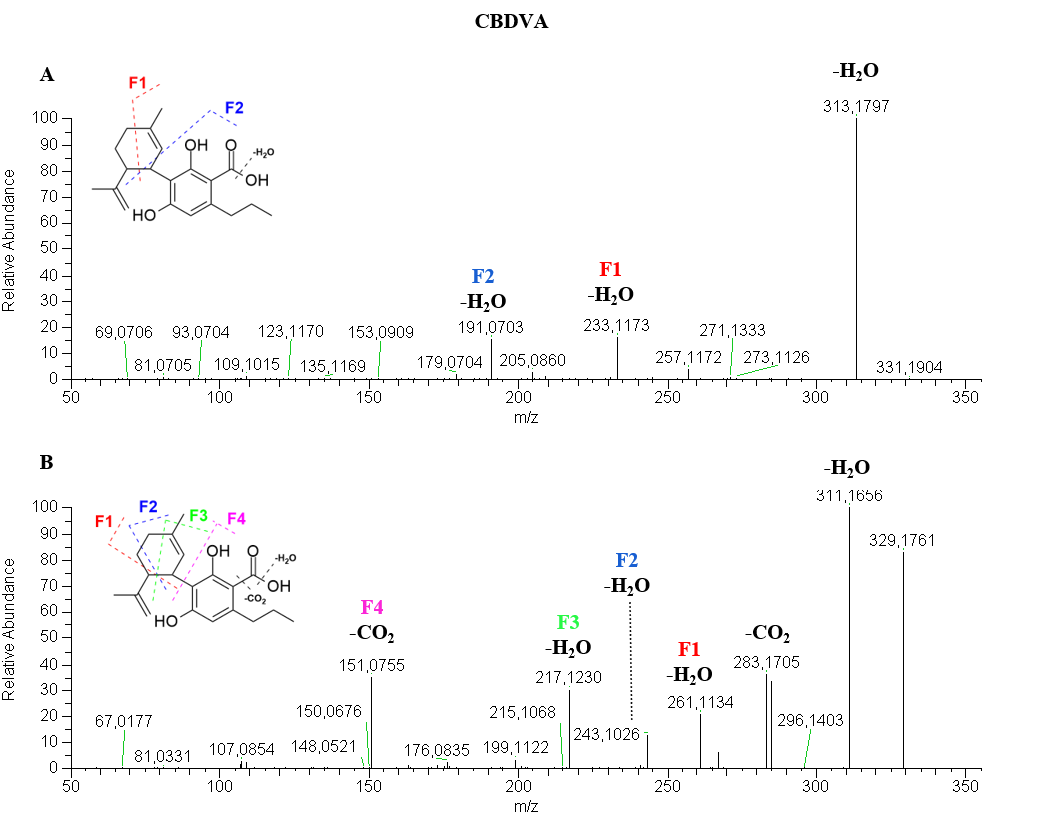


Figure S 5. HRMS fragmentation spectrum of cannabidivarinic acid (CBDVA) in positive (A) and negative (B) ionization mode.


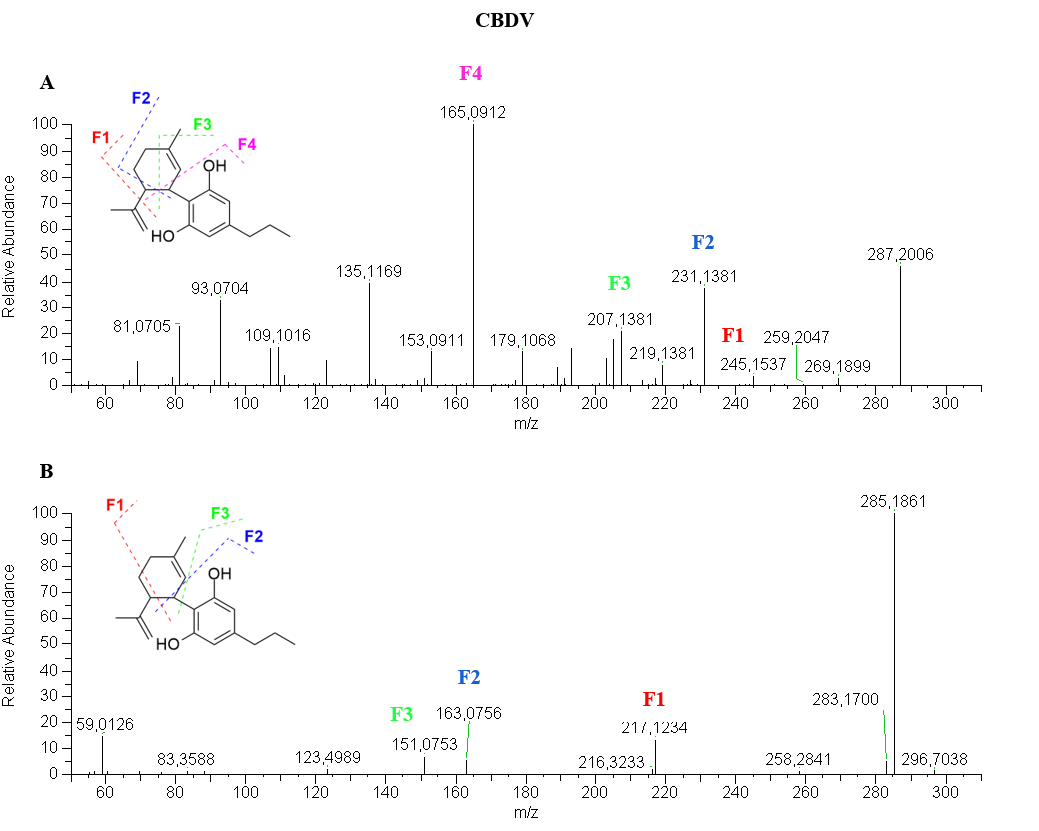


Figure S 6. HRMS fragmentation spectrum of cannabidivarin (CBDV) in positive (A) and negative (B) ionization mode.


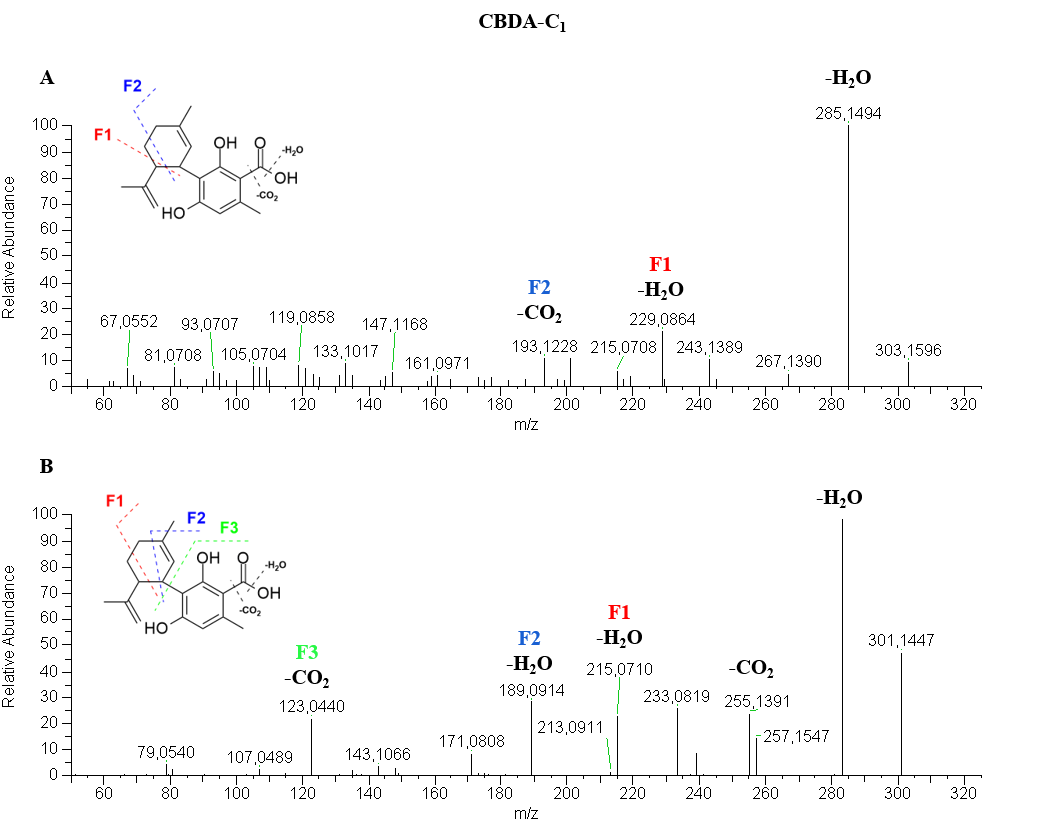


Figure S 7. HRMS fragmentation spectrum of cannabidiolic acid-C_1_ (CBDA-C_1_) in positive (A) and negative (B) ionization mode.


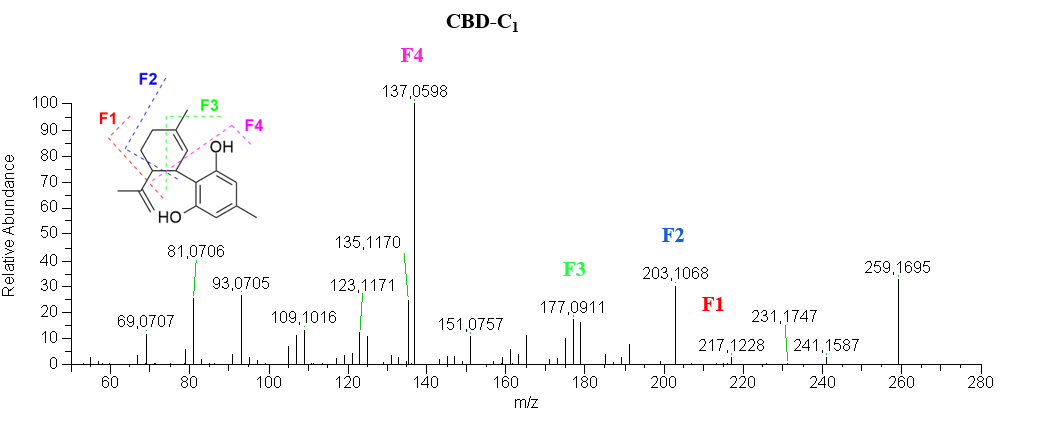


Figure S 8. HRMS fragmentation spectrum of cannabidiol-C_1_ (CBD-C_1_) in positive ionization mode.


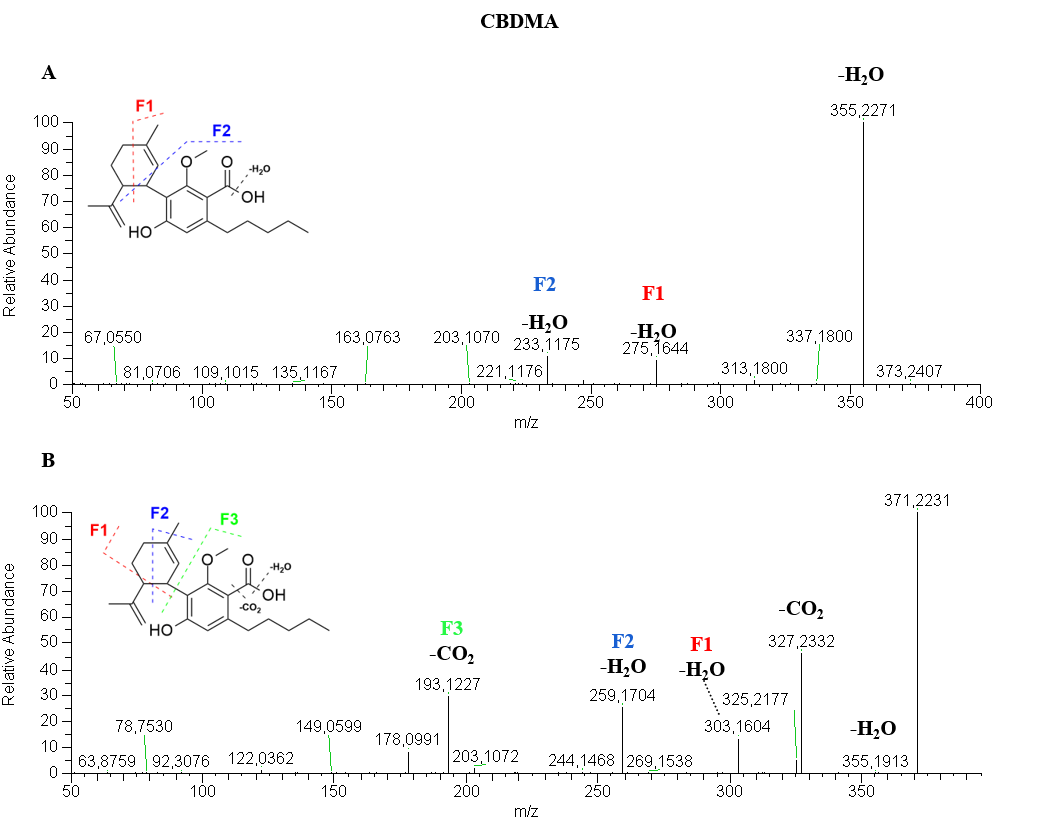


Figure S 9. HRMS fragmentation spectrum of cannabidiolic acid monomethyl ether (CBMA) in positive (A) and negative (B) ionization mode.


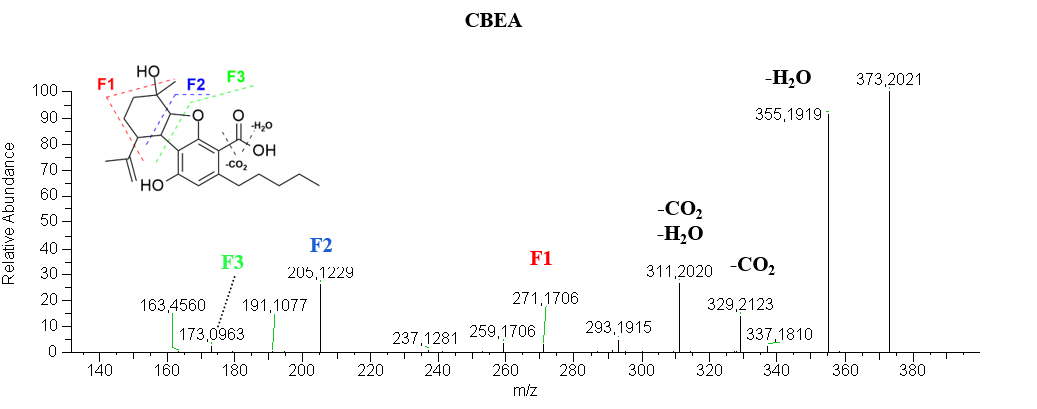


Figure S 10. HRMS fragmentation spectrum of cannabielsoinic acid (CBEA) in negative ionization mode.


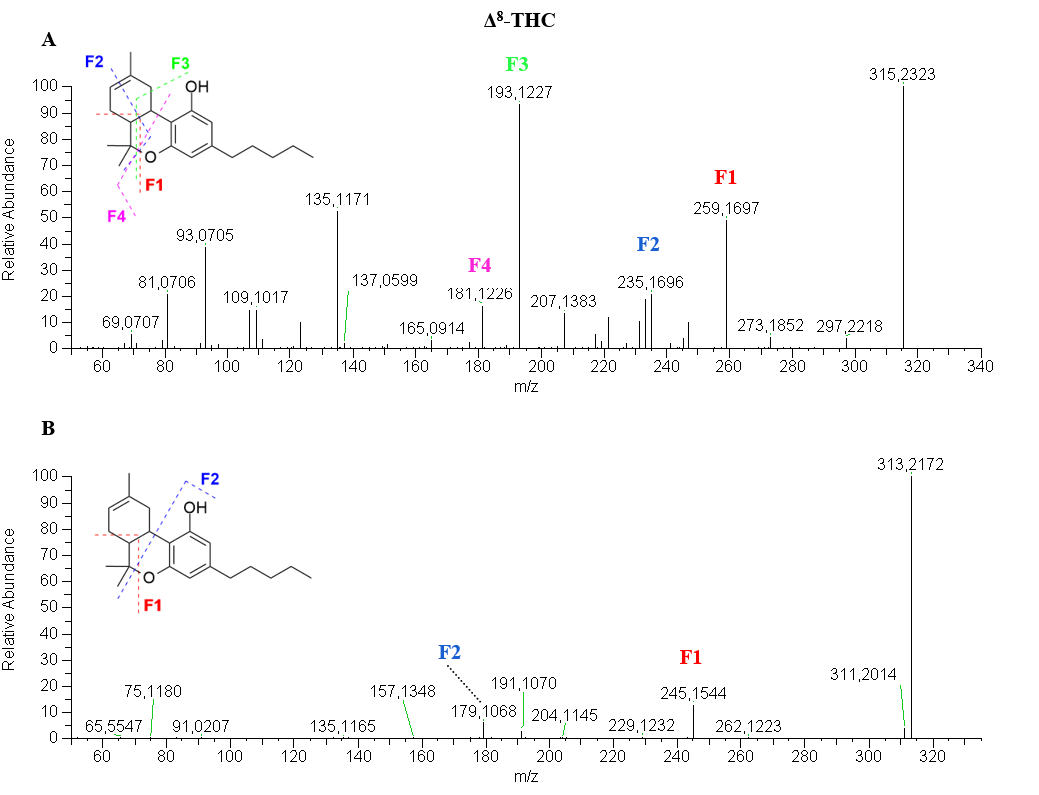


Figure S 11. HRMS fragmentation spectrum of Δ^8^-tetrahydrocannabinol (Δ^8^-THC) in positive (A) and negative (B) ionization mode.


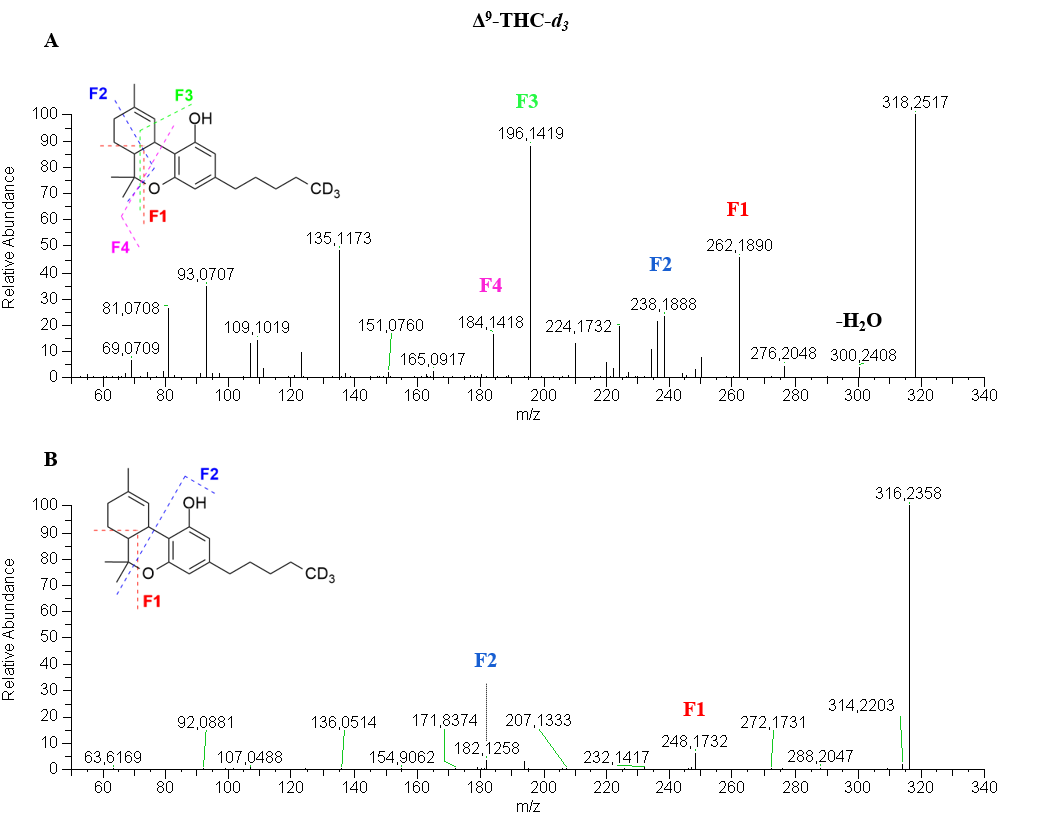


Figure S 12. HRMS fragmentation spectrum of Δ^9^-tetrahydrocannabinol-*d_3_* (Δ^9^-THC-*d_3_* or THC-*d_3_*) in positive (A) and negative (B) ionization mode.


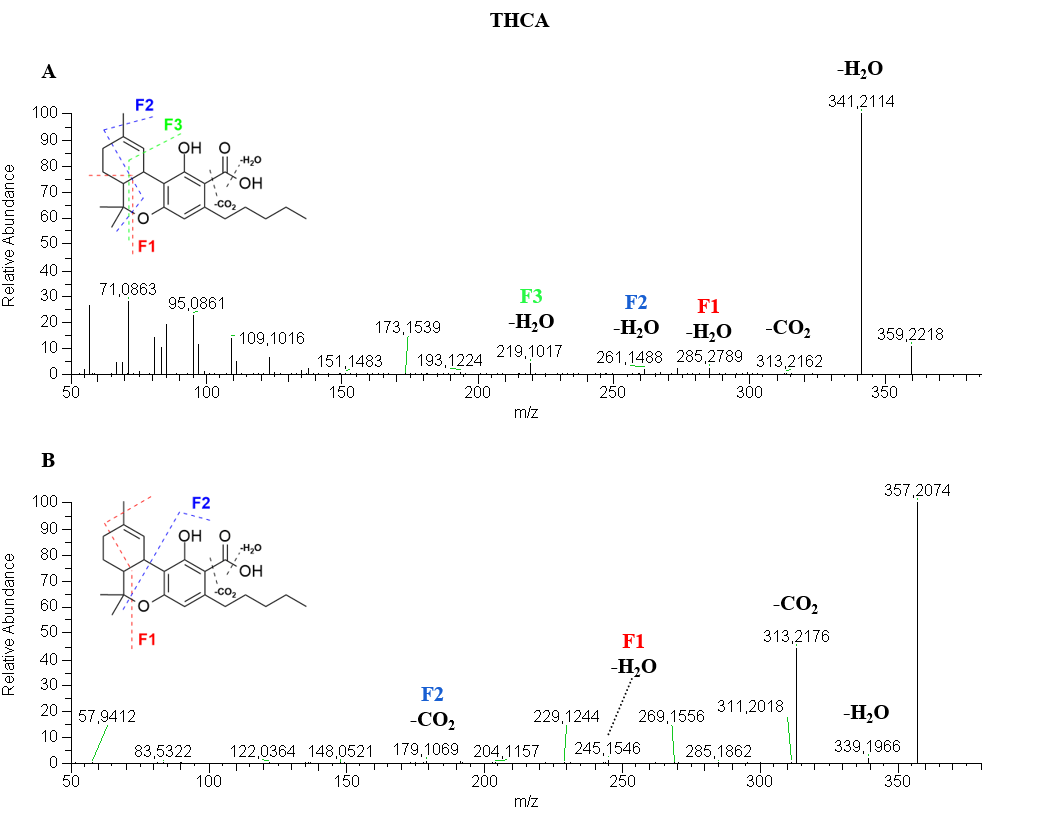


Figure S 13. HRMS fragmentation spectrum of tetrahydrocannabinolic acid (THCA) in positive (A) and negative (B) ionization mode.


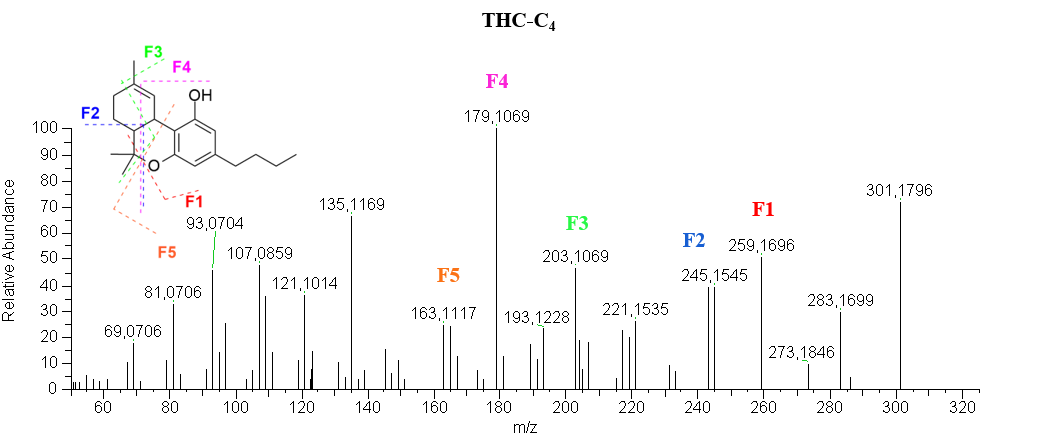


Figure S 14. HRMS fragmentation spectrum of tetrahydrocannabinol-C_4_ (THC-C_4_) in positive ionization mode.


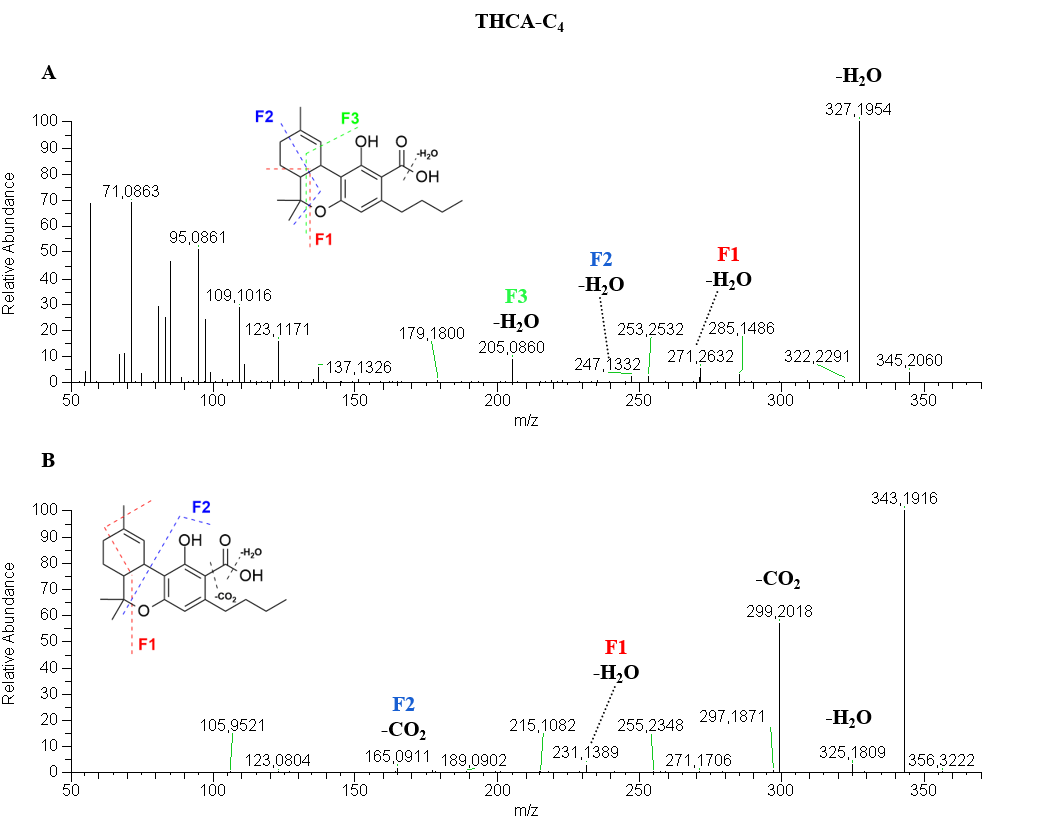


Figure S 15. HRMS fragmentation spectrum of tetrahydrocannabinolic acid-C_4_ (THCA-C_4_) in positive (A) and negative (B) ionization mode.


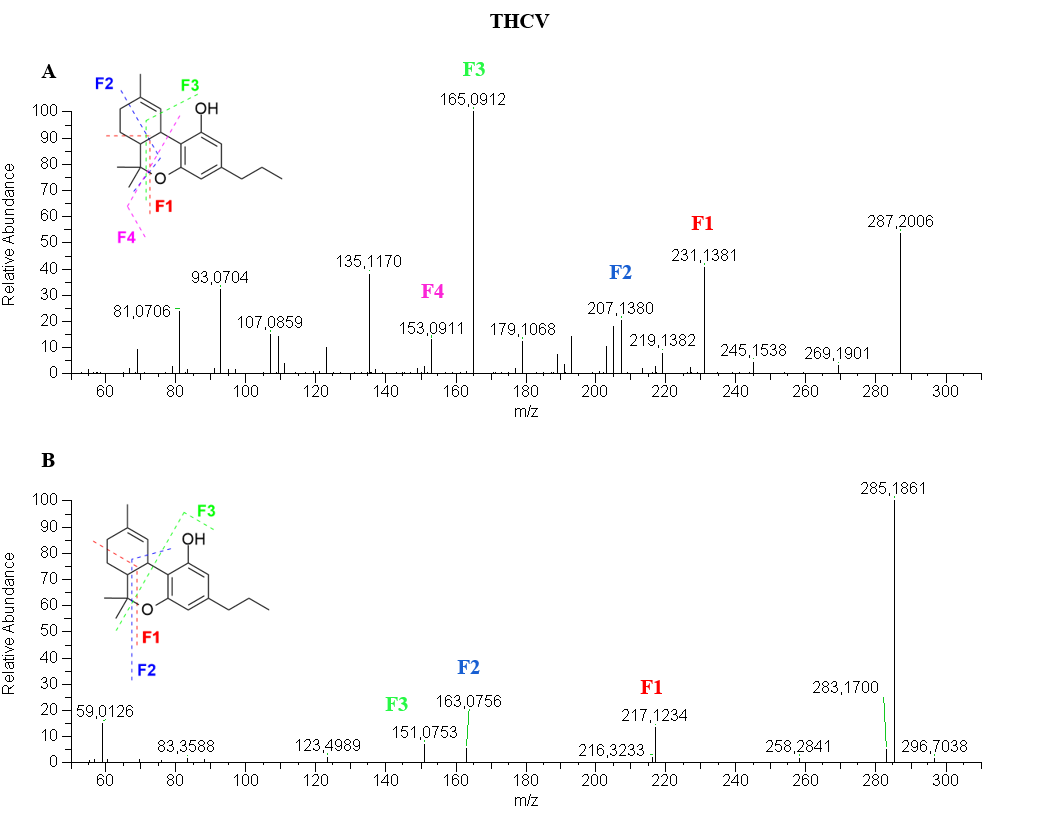


Figure S 16. HRMS fragmentation spectrum of tetrahydrocannabidivarin (THCVA) in positive (A) and negative (B) ionization mode.


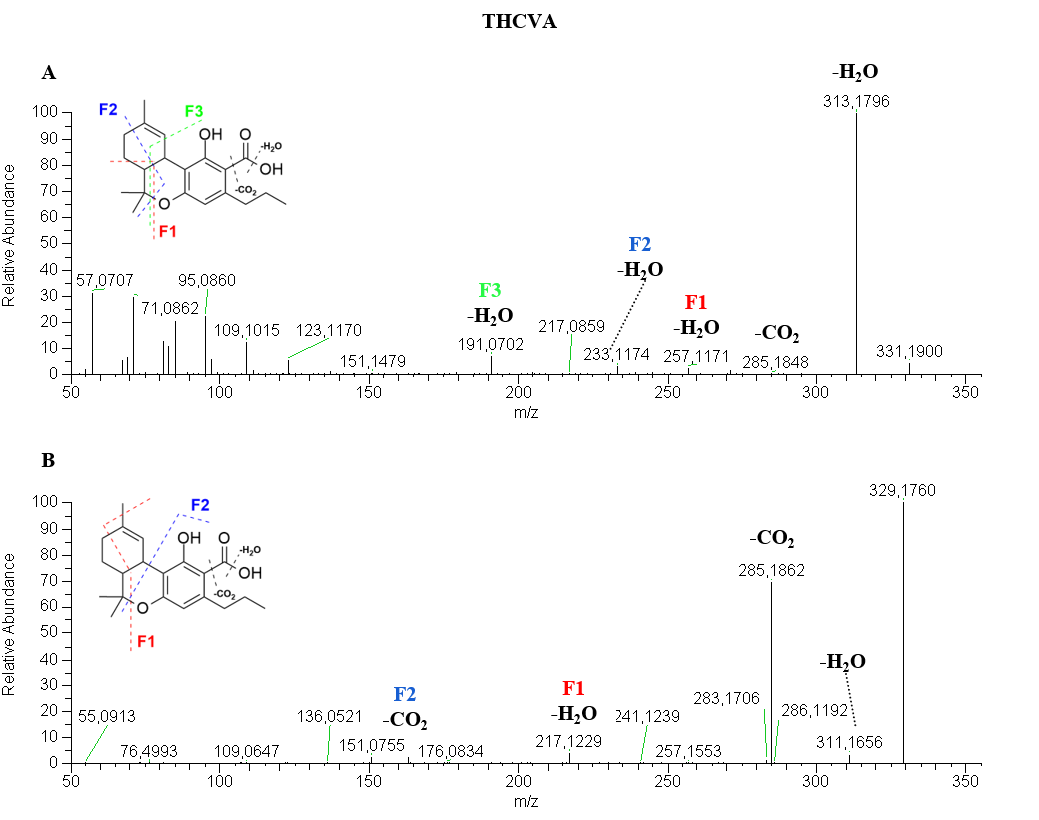


Figure S 17. HRMS fragmentation spectrum of tetrahydrocannabidivarinic acid (THCVA) in positive (A) and negative (B) ionization mode.


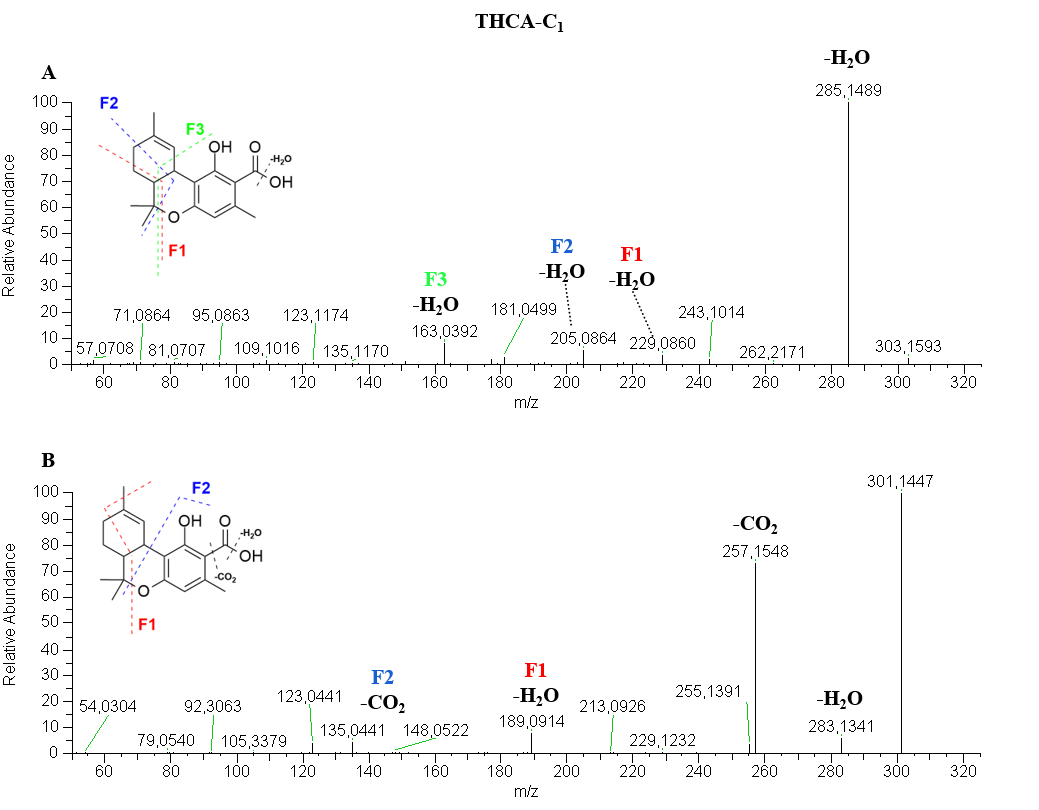


Figure S 18. HRMS fragmentation spectrum of tetrahydrocannabinolic acid-C_1_ (THCA-C_1_) in positive (A) and negative (B) ionization mode.


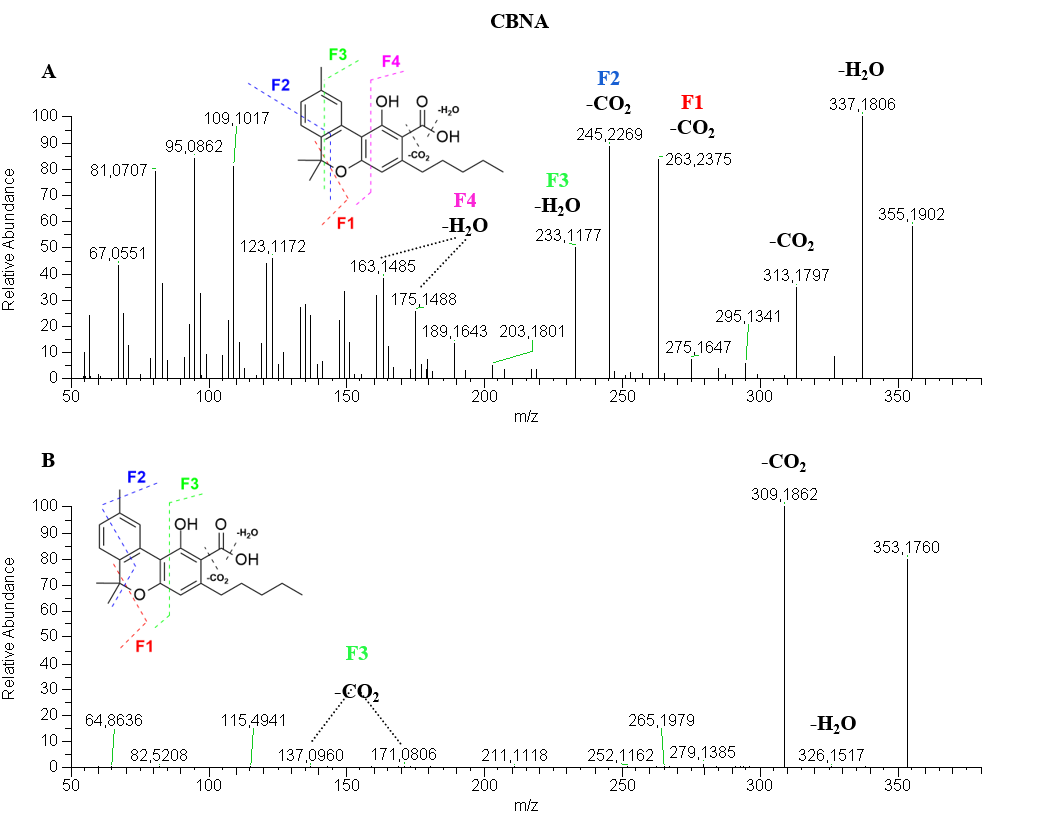


Figure S 19. HRMS fragmentation spectrum of cannabinolic acid (CBNA) in positive (A) and negative (B) ionization mode.


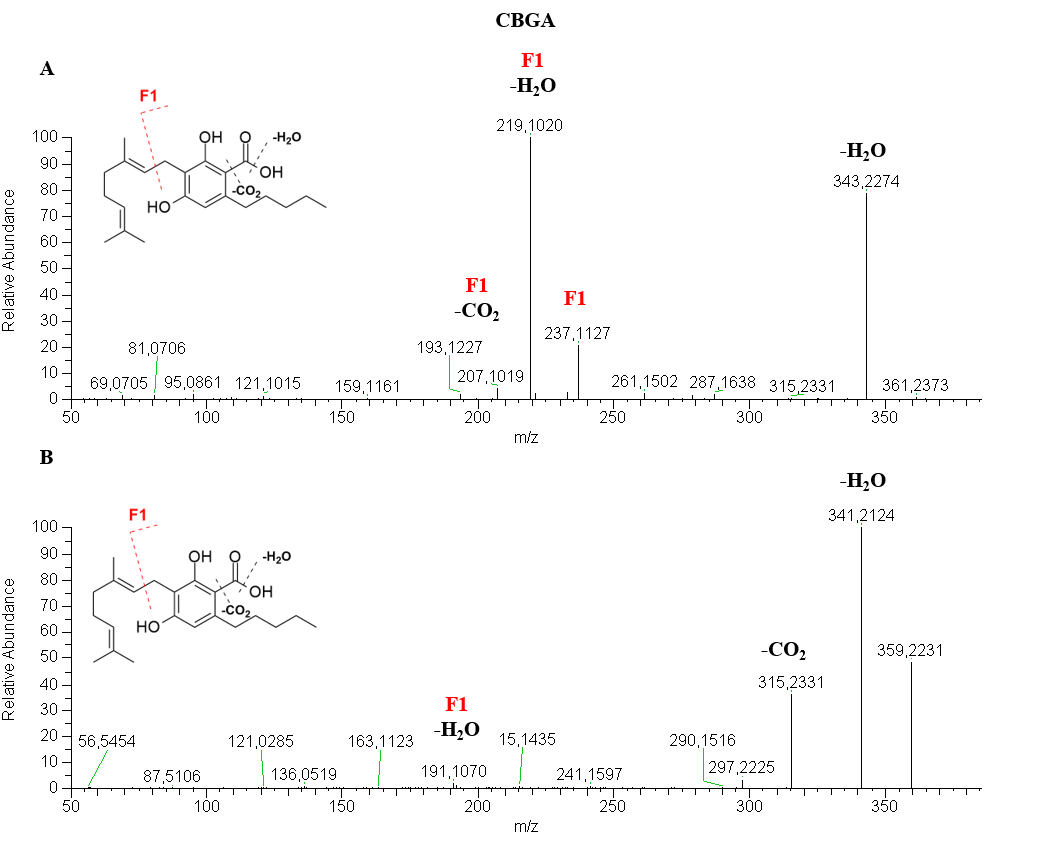


Figure S 20. HRMS fragmentation spectrum of cannabigerolic acid (CBGA) in positive (A) and negative (B) ionization mode.


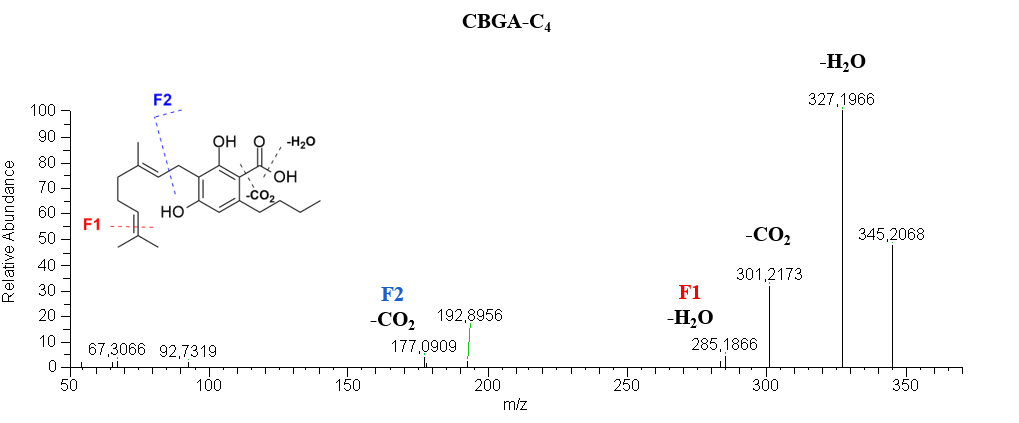


Figure S 21. HRMS fragmentation spectrum of cannabigerolic acid-C_4_ (CBGA-C_4_) in negative ionization mode.


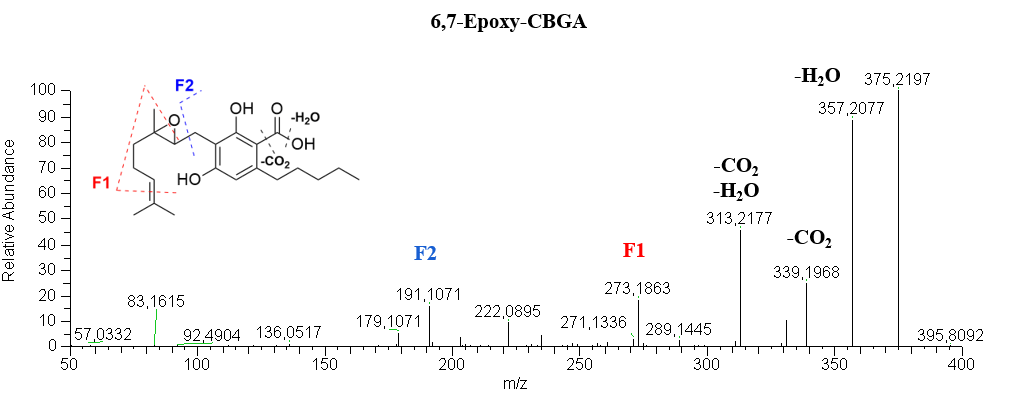


Figure S 22. HRMS fragmentation spectrum of 6,7-epoxy-cannabigerolic acid (6,7-epoxy-CBGA) in negative ionization mode.


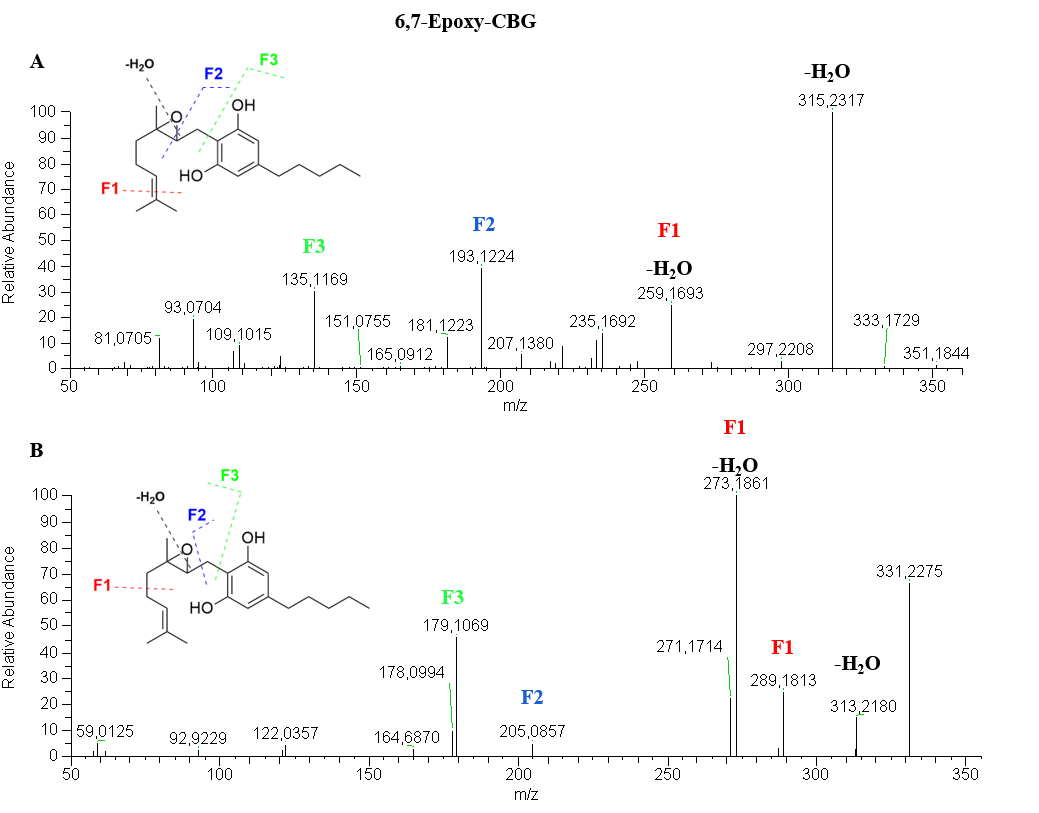


Figure S 23. HRMS fragmentation spectrum of 6,7-epoxy-cannabigerol (6,7-epoxy-CBG) in positive (A) and negative (B) ionization mode.


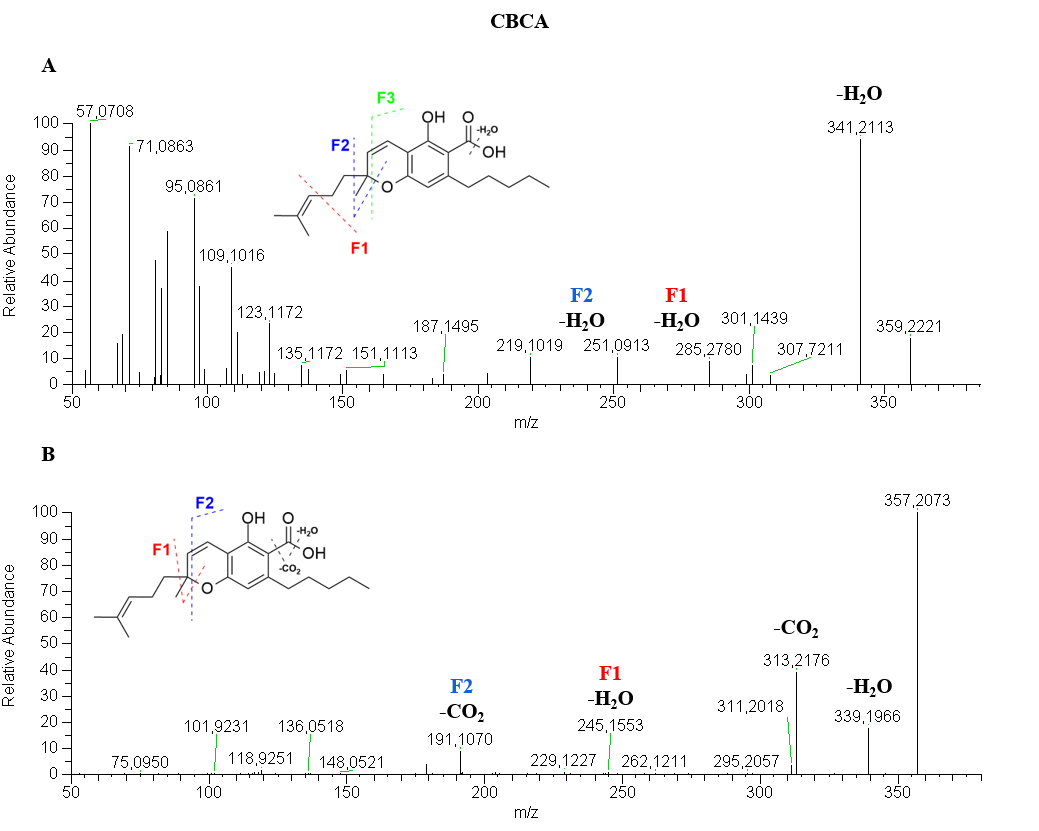


Figure S 24. HRMS fragmentation spectrum of cannabichromenic acid (CBCA) in positive (A) and negative (B) ionization mode.


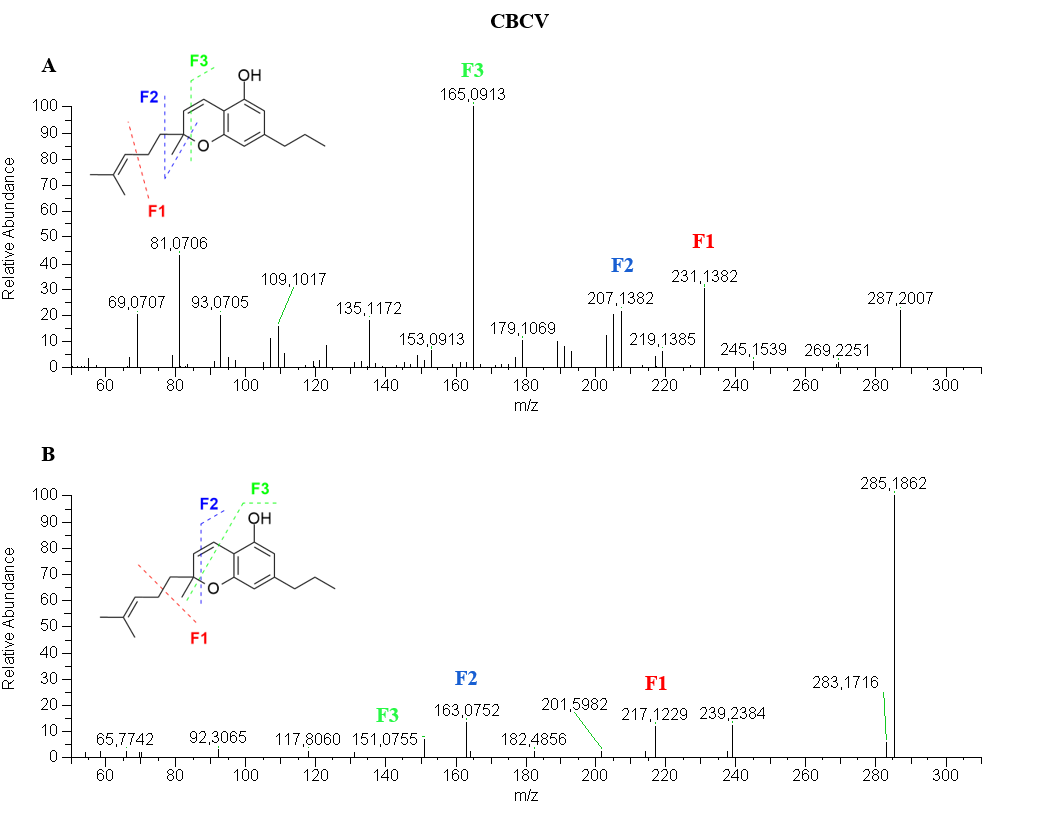


Figure S 25. HRMS fragmentation spectrum of cannabichromevarin (CBCV) in positive (A) and negative (B) ionization mode.


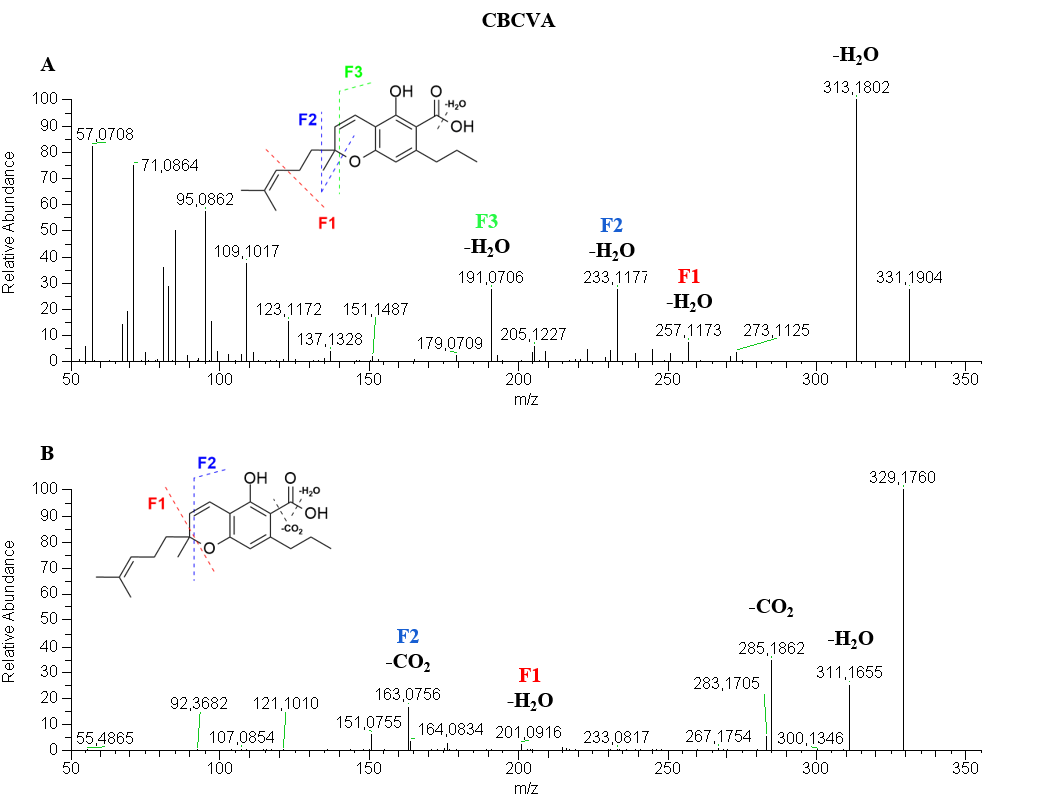


Figure S 26. HRMS fragmentation spectrum of cannabichromevarinic acid (CBCVA) in positive (A) and negative (B) ionization mode.


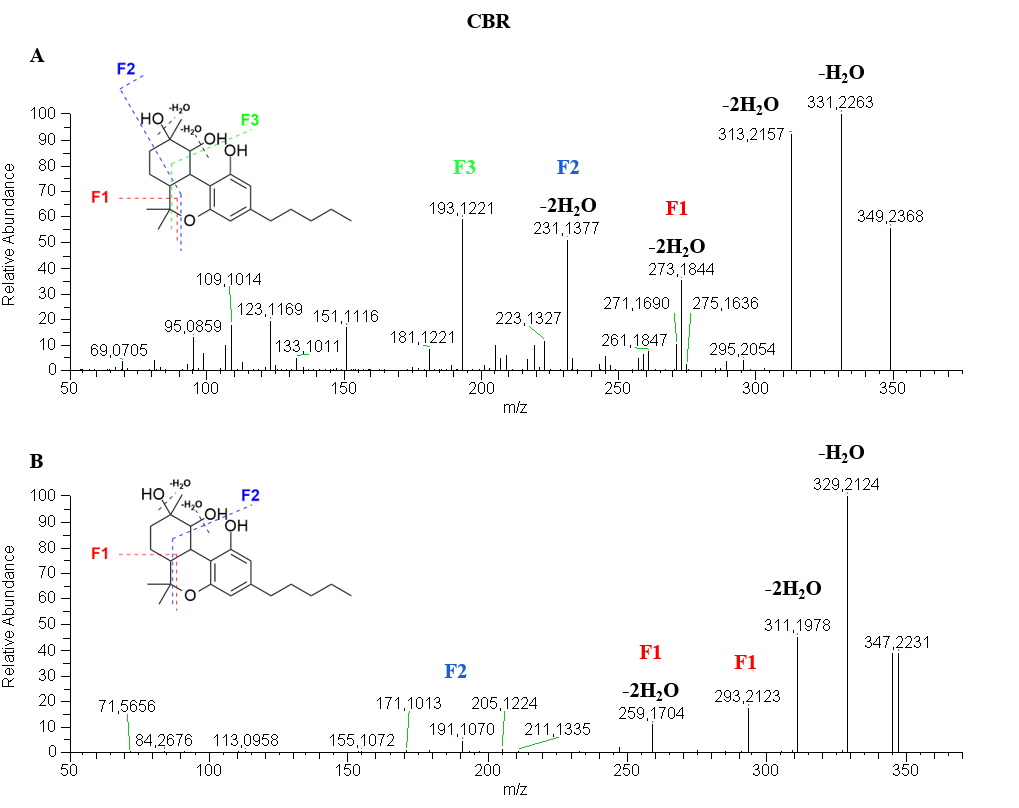


Figure S 27. HRMS fragmentation spectrum of cannabiripsol (CBR) in positive (A) and negative (B) ionization mode.


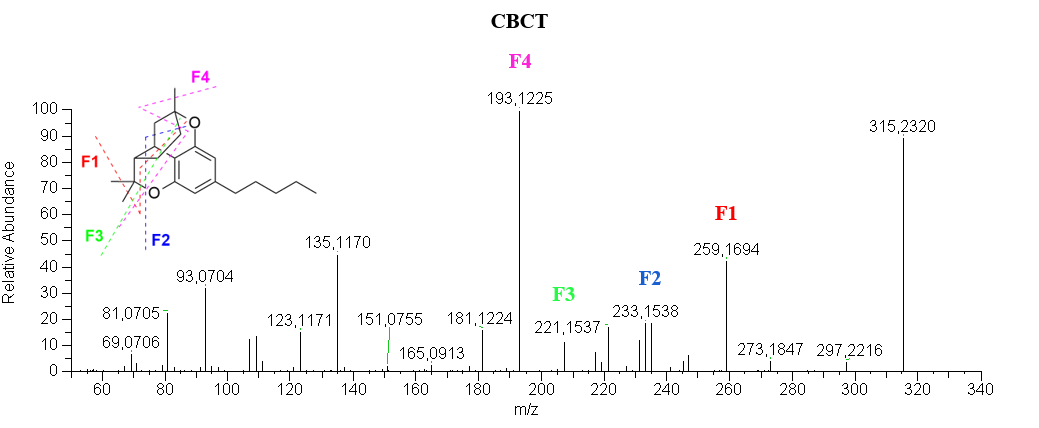


Figure S 28. HRMS fragmentation spectrum of cannabicitran (CBCT) in positive ionization mode.


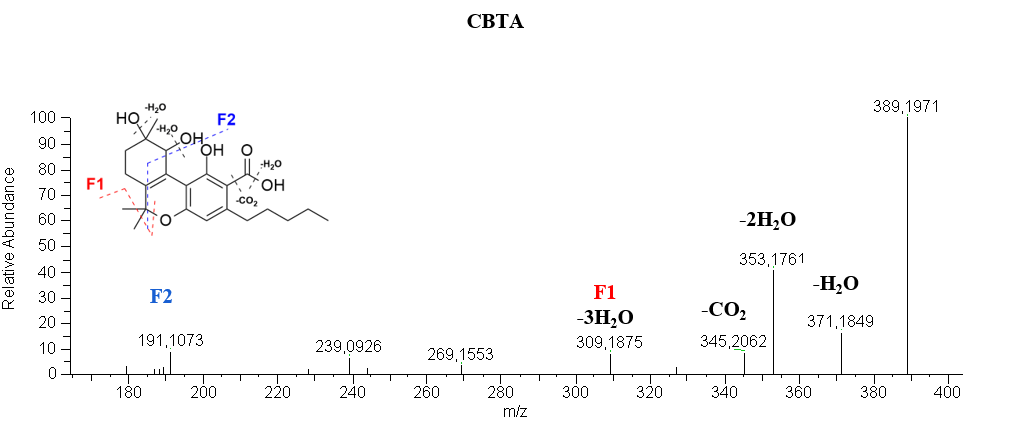


Figure S 29. HRMS fragmentation spectrum of cannabitriolic acid (CBTA) in negative ionization mode.


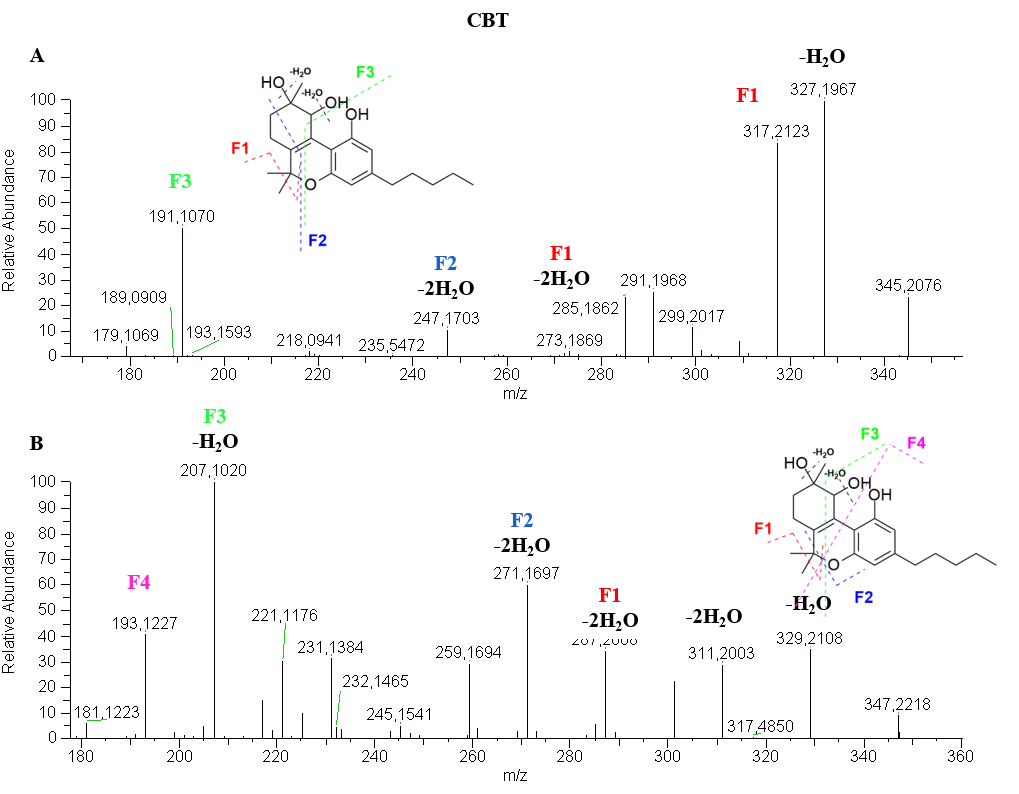


Figure S 30. HRMS fragmentation spectrum of cannabitriol (CBT) in positive (A) and negative (B) ionization mode.

**F4**

**F3**

**F2**

**F1**

**F3**

## Multivariate Statistical Analysis


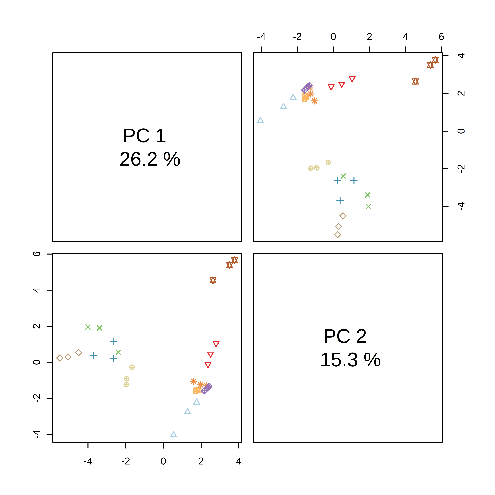


Figure S 31. PCA overview for two principal components.

**
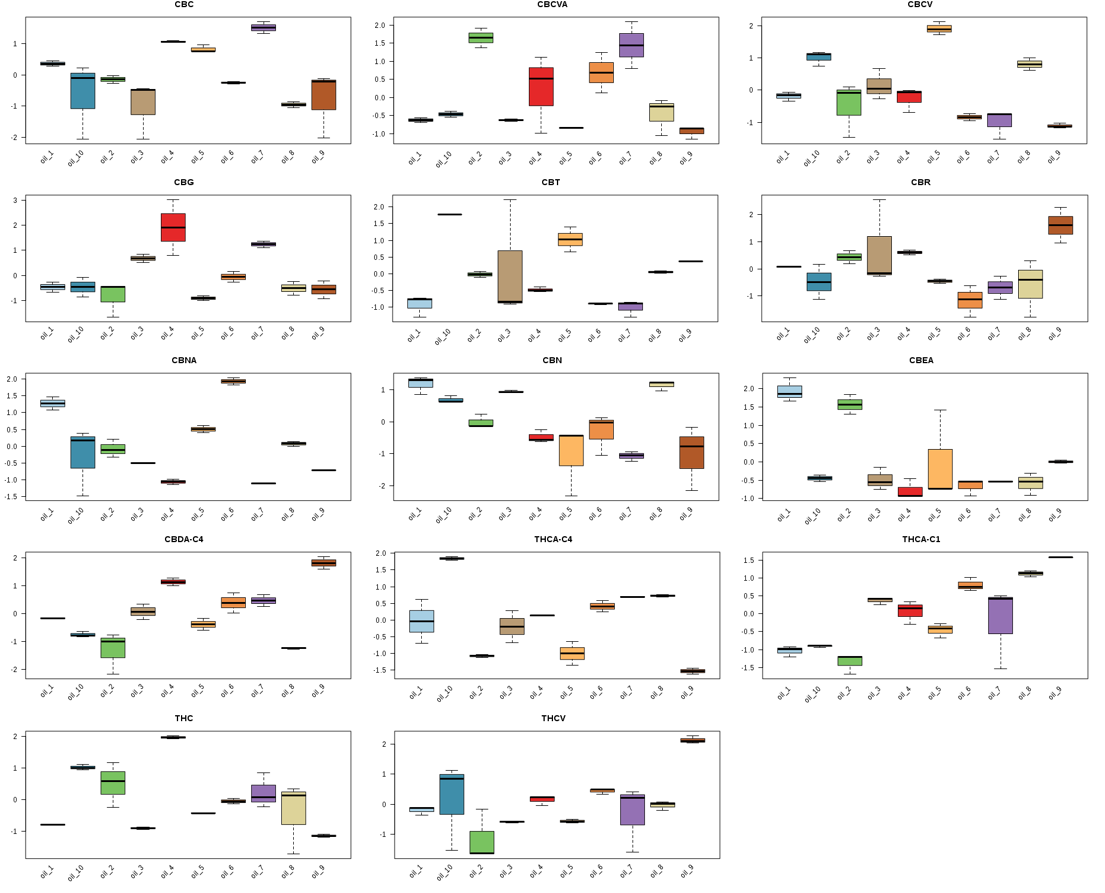
**

Figure S 32. Box-and-whisker plots of the 22 statistically significant cannabinoids extracted from one-way ANOVA test. The distribution of each cannabinoid in the ten samples is represented as median with error bars (variability).

## Semi-quantification

Table S1. Calibration curves of cannabinoid analytical standards for the semi-quantification of cannabinoids identified in hemp seed oil. Calibration curves were built with seven non-zero points in the range 0.01-1.00 µg/mL. Each calibration point was analyzed three times.

| **Compound** | **Calibration curve** | **R^2^** | **Reference standard** |
| --- | --- | --- | --- |
| CBGA | y=1E09x+4E06 | 0.9949 | CBGA |
| CBG | y=3E08x-4E06 | 0.9984 | CBG |
| CBDA | y=1E09x+7E07 | 0.9976 | CBDA |
| CBD | y=8E08x+1E07 | 0.9978 | CBD |
| CBDV | y=6E07x-357705 | 0.9992 | CBDV |
| CBC | y=1E08x+5E06 | 0.9927 | CBC |
| THCA | y=1E08x+1E07 | 0.9940 | THCA |
| Δ^9^-THC | y=1E09x+3E07 | 0.9964 | Δ^9^-THC |
| CBGA-C_4_, 6,7-epoxy-CBGA | y=1E09x+4E06 | 0.9949 | CBGA |
| 6,7-Epoxy-CBG | y=3E08x-4E06 | 0.9984 | CBG |
| CBN | y=1E08x-557975 | 0.9984 | CBN |
| CBDA-C_4_, CBDVA, CBDA-C_1_, CBDMA | y=1E09x+7E07 | 0.9976 | CBDA |
| CBD-C_4_, CBD-C_1_ | y=8E08x+1E07 | 0.9978 | CBD |
| THCA-C_4_, THCVA, THCA-C_1_ | y=1E08x+1E07 | 0.9940 | THCA |
| THC-C_4_, THCV | y=1E09x+3E07 | 0.9964 | Δ^9^-THC |
| CBCA, CBCVA, CBNA, CBEA, CBTA | y=7E08x+3E07 | 0.9998 | Av. CBGA-THCA-CBDA |
| CBT, CBR, CBCT | y=6E08x+1E07 | 0.9978 | Av. CBD, Δ^9^-THC, CBG, CBC |
